# Live-Cell Fluorescence Lifetime Multiplexing Using Synthetic Fluorescent Probes

**DOI:** 10.1021/acschembio.2c00041

**Published:** 2022-05-18

**Authors:** Michelle S. Frei, Birgit Koch, Julien Hiblot, Kai Johnsson

**Affiliations:** † Department of Chemical Biology, Max Planck Institute for Medical Research, Jahnstrasse 29, 69120 Heidelberg, Germany; ‡ Institute of Chemical Sciences and Engineering (ISIC), École Polytechnique Fédérale de Lausanne (EPFL), 1015 Lausanne, Switzerland

## Abstract

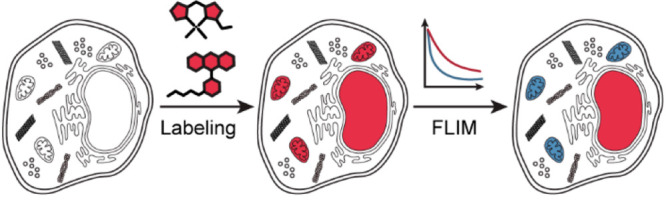

Fluorescence lifetime multiplexing requires fluorescent probes with distinct fluorescence lifetimes but similar spectral properties. Even though synthetic probes for many cellular targets are available for multicolor live-cell fluorescence microscopy, few of them have been characterized for their use in fluorescence lifetime multiplexing. Here, we demonstrate that, from a panel of 18 synthetic probes, eight pairwise combinations are suitable for fluorescence lifetime multiplexing in living mammalian cell lines. Moreover, combining multiple pairs in different spectral channels enables us to image four and with the help of self-labeling protein tags up to eight different biological targets, effectively doubling the number of observable targets. The combination of synthetic probes with fluorescence lifetime multiplexing is thus a powerful approach for live-cell imaging.

Fluorescence microscopy is an indispensable tool to noninvasively investigate dynamic processes in living cells. Such experiments often require imaging multiple biomolecules and cellular compartments simultaneously. This is generally achieved by spectrally resolved detection using fluorophores with distinct excitation and emission spectra (Figure 1A). However, even though fluorophores that cover the entire visible spectrum are available,^1,2^ this approach is often limited to three to four channels as the spectra of the fluorophores overlap.^3^ Strategies to expand the degree of multiplexing have not only centered on techniques to improve spectral imaging^4−6^ but also make use of other fluorophore properties to access higher dimensions. One such property is fluorescence lifetime, which has been used for multiplexing via fluorescence lifetime imaging microscopy (FLIM, Figure 1B).^7,8^ Recently, fluorescence lifetime multiplexing was combined with spectral multiplexing (S-FLIM) to further increase the number of simultaneously observable targets.^9,10^

**Figure 1:**
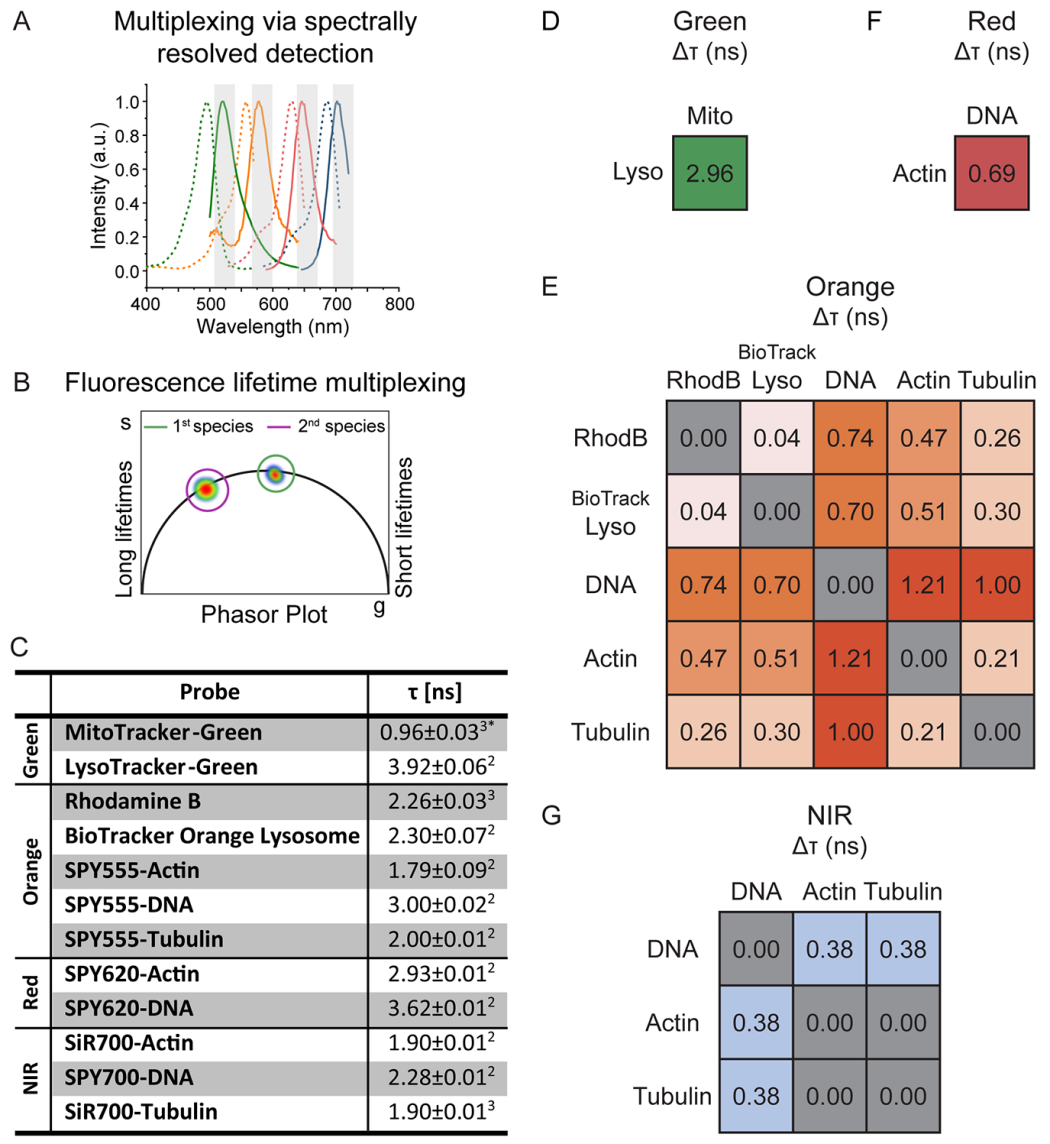
Fluorescence lifetime characterization of synthetic probes. (A, B) Schematic representation of multiplexing via spectrally resolved detection (A) or fluorescence lifetime multiplexing (B). (C) Average intensity weighted fluorescence lifetime (τ) of the 12 probes suitable for fluorescence lifetime multiplexing. Mean ± SEM, *N* = 4 field of views from two biological replicates. (2) biexponential fit, (3) triexponential fit, (*) tail fit (all others n-exponential reconvolution fit). (D–G) Differences in average intensity weighted fluorescence lifetime (Δτ) between probes in the green (D), orange (E), red (F), and NIR (D) spectral region.

Synthetic probes based on small-molecule fluorophores for live-cell microscopy of various subcellular targets such as lysosomes, mitochondria, or filamentous actin (F-actin) are available.^11^ They do not require genetic engineering (e.g., transfection) of the target cell and can therefore be applied to a wide variety of cell types. Additionally, probes for different targets can easily be combined, while the simultaneous expression of multiple tagged proteins can be challenging.^12^ Indeed, synthetic probes with distinct spectral properties were successfully combined for multiplexing.^13−15^ However, they have only found limited use in fluorescence lifetime multiplexing, and their fluorescence lifetimes are often not characterized.^9,10,16,17^ Proof-of-concept studies were restricted to fixed cell applications^9,10^ and/or used probes with both differences in fluorescence lifetime and emission spectrum.^9,10,16^ We recently demonstrated the combined use of synthetic probes and self-labeling protein tags for fluorescence lifetime multiplexing.^17^ However, our study was limited to only four probes and thus only partially exploited the potential of synthetic probes for fluorescence lifetime multiplexing.

Here, we investigate if fluorophores of different classes and chemically identical fluorophores targeted to different subcellular localizations show differences in fluorescence lifetime. Indeed, internal and external factors including vibrational and rotational freedom, viscosity, polarity, or the presence of quenching moieties can influence the fluorescence lifetime of fluorophores.^18^ Combinations of live-cell compatible, synthetic probes for biomolecules or cellular compartments could find applications in live-cell fluorescence lifetime multiplexing. Ideally, each probe should show a homogeneous and narrow fluorescence lifetime distribution, and spectrally similar probes should show differences in fluorescence lifetimes to enable their separation.

We therefore characterized the spectral and fluorescence lifetime properties of 18 commercially available cell permeable probes. These encompassed popular rhodamine and BODIPY based probes in five different spectral channels targeting DNA, F-actin, microtubules, mitochondria, and lysosomes (Supporting Table S1).^13,15,19,20^ Four probes were previously used for fluorescence lifetime multiplexing,^16,17^ and another two were used in spectral FLIM (S-FLIM).^9,10^ For initial screening purposes we choose to assess the fluorescence lifetime properties by phasor analysis^21,22^ as it allows rapid and visual screening of probes for differences in fluorescence lifetime without the need for fitting (Figure 1B, Supporting Figure S1). Specifically, we labeled living U-2 OS cells with all 18 probes individually and acquired FLIM images. Phasor plot analysis then revealed narrow and homogeneous distributions for all actin, microtubule, and DNA probes. Probes for lysosomes had slightly broader distributions but were still homogeneous. However, only two of the four probes for mitochondria displayed satisfying properties (MitoTracker-Green and rhodamine B). The other two, MitoTracker-Red and MitoTracker-Orange, showed multiple fluorescence lifetime populations in their phasor plots (Supporting Figure S1). This might result from the probes accumulating in multiple organelles as previously reported for MitoTracker-Orange in fixed cells (mitochondria, nucleoli, and endosome).^9^ While these differences in fluorescence lifetime might be used to separate the specific mitochondria signal from the unspecific signal in the endosome and the nucleoli, these probes cannot be combined with other spectrally similar probes for further fluorescence lifetime multiplexing, and hence they were not further investigated.

Next, we assessed the differences in fluorescence lifetime within one spectral channel by overlaying the measured phasor plots of the pure species. If differences were found, the individual average intensity weighted fluorescence lifetime was quantified by curve fitting. This then allowed the calculation of the fluorescence lifetime differences between probes in the same spectral channel. The largest differences in fluorescence lifetime were found in the green spectral channel between LysoTracker-Green (3.92 ± 0.06 ns) and MitoTracker-Green (0.96 ± 0.03 ns) followed by SPY555-DNA (3.00 ± 0.02 ns) and SPY555-Actin (1.79 ± 0.09 ns) in the orange channel.

These two pairs are hence ideally suited for fluorescence lifetime multiplexing (Figure 1C–E). Differences were also found for probes in the red and NIR spectral region (Figure 1F,G). Probes based on the popular silicon rhodamine (SiR) did not show any differences in fluorescence lifetime and were therefore not further investigated (Supporting Figure S1). Generally, probes based on different fluorophore scaffolds (e.g., BODIPY vs rhodamine) showed the biggest differences in fluorescence lifetime. The excitation and emission spectra of probes within one spectral region were generally highly similar, except for LysoTracker-Red, which shows a 20 nm hypsochromic shift in comparison to the two SPY620 probes (Supporting Figure S2). LysoTracker-Red was therefore not considered for multiplexing experiments. Furthermore, we demonstrated that most probes’ fluorescence lifetime showed only little variation between different cell lines (HeLa and HEK293, Supporting Table S2). An exception are the lysosome probes, which generally showed broad fluorescence lifetime distributions (Supporting Figure S1) and therefore higher variability in average intensity weighted fluorescence lifetime within one cell line as well as between different cell lines. This variability might stem from differences in the intralysosomal pH.^23^ Further, we characterized the crosstalk for different probe combinations using the images from individually labeled U-2 OS cells (Supporting Figure S3, Supporting Table S3). Most probe combinations showed less than 10% crosstalk into the other species’ lifetime channel. The combination BioTracker Orange Lysosome (11.7 ± 4.7%)–SPY555-DNA (14.2 ± 1.6%) had the biggest crosstalk.

We then performed fluorescence lifetime multiplexing using pairwise combinations of two probes in each of the four spectral regions (green: 489 nm excitation, 510–540 nm emission; orange: 550 nm excitation, 570–600 nm emission; red: 615 nm excitation, 635–700 nm emission; near-infrared (NIR): 670 nm excitation, 710–760 nm emission). As predicted by the differences in average intensity weighted fluorescence lifetime (Figure 1D–G), multiple combinations of probes could be separated using fluorescence lifetime information. Suitable pairs were found in all four spectral regions (Figure 2A–D). For instance, it was possible to image mitochondria and lysosomes simultaneously using LysoTracker-Green and MitoTracker-Green in the green channel (Figure 2A). The nucleus and F-actin can be separated using probes in either the orange, the red, or the NIR channel (Figure 2C, Supporting Figures S4, S5). Moreover, average photon arrival times as reported in the FastFLIM image could be used to distinguish the two species if they are spatially separated and to identify overlapping regions (pixels with intermediate fluorescence lifetime). This allows for quick inspection during image acquisition without the need for fitting or phasor analysis, greatly simplifying the use of fluorescence lifetime multiplexing. As the probes’ fluorescence lifetimes showed little variation between cell types (Supporting Table S2), multiplexing could also be performed in living HEK 293 or HeLa cells (Supporting Figure S6).

**Figure 2:**
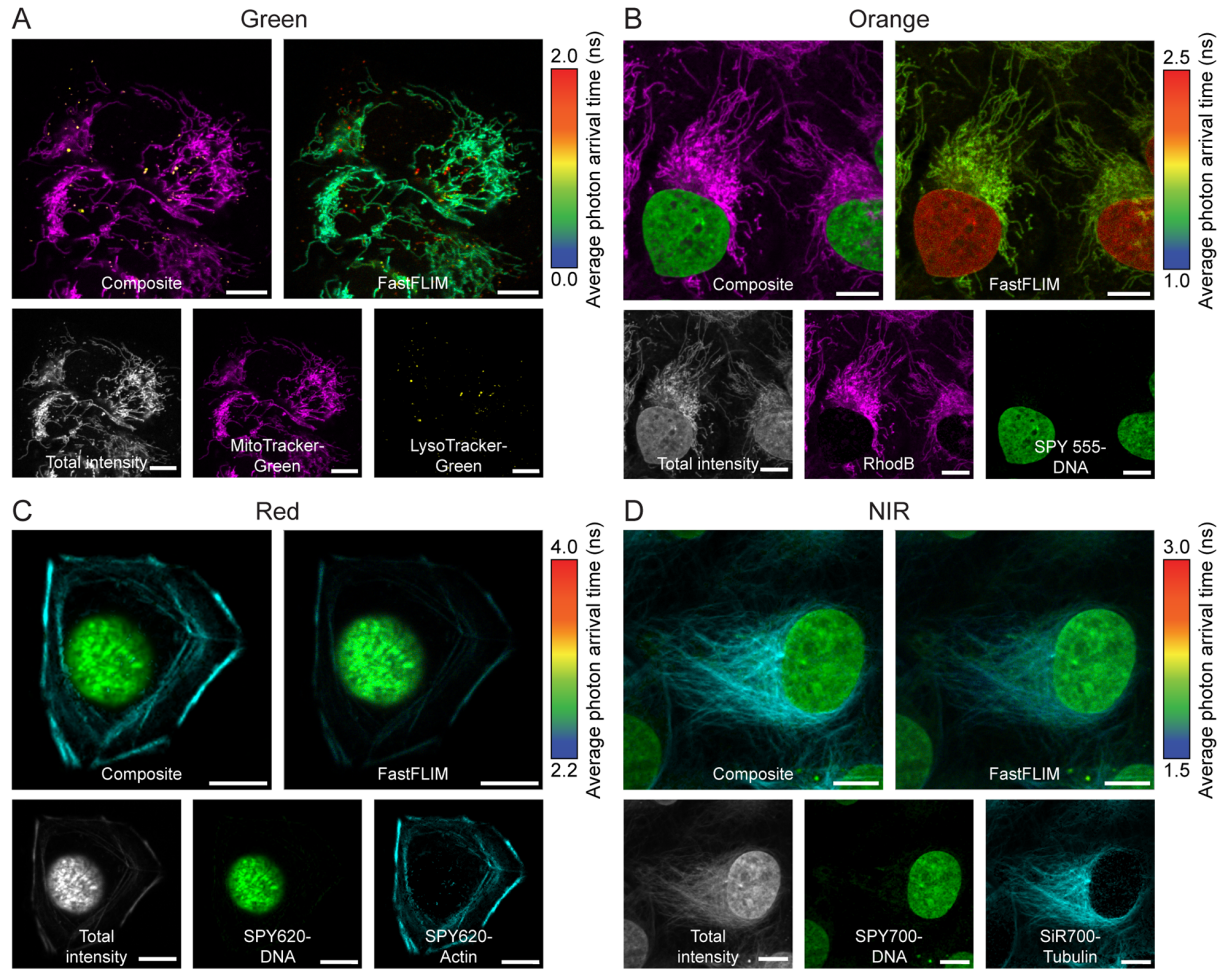
Live-cell fluorescence lifetime multiplexing in four different spectral regions. (A–D) U-2 OS cells were labeled with MitoTracker-Green and LysoTracker-Green (A), Rhodamine B and SPY555-DNA (B), SPY620-DNA and SPY620-Actin (C), or SPY700-DNA and SiR700-Tubulin (D) and imaged by FLIM. In each spectral channel, the two species could be clearly separated based on fluorescence lifetime information. The FastFLIM image reports the average photon arrival time and allows for quick visual inspection of the two species. The composite, the FastFLIM image with the respective color scale, the total fluorescence intensity, and the two individual separated species are given. Species separation was achieved using the phasor approach (positioning the cluster circles on the phasor plot at the position of the pure species). Scale bars, 10 μm.

In general, larger differences in fluorescence lifetime and narrow fluorescence lifetime distributions of the individual species facilitate separation especially if the two species exhibit overlapping regions. We therefore recommend the use of pairs of probes with a fluorescence lifetime difference of around 0.5 ns. Species with smaller differences (e.g., SiR700-Tubulin and SPY700-DNA) can still be separated, but crosstalk becomes more important, and it is hence important to choose probes with narrow fluorescence lifetime distributions. Separation is furthermore influenced by the brightness of the two species: to obtain comparable signal-to-background ratios for both species after separation, similar photon numbers should be collected. Otherwise, the crosstalk of the brighter species into the dimmer species lifetime channel will approach the dimmer species’ signal intensity. Acquiring similar photon numbers in FLIM can be challenging when working with probes of different brightness as the fluorescence of both probes is acquired simultaneously using only one excitation wavelength. However, the *in cellulo* brightness of synthetic probes is not only determined by their molecular brightness but can also be adjusted through the degree/density of labeling and hence the labeling concentration. Specifically, mitochondria and lysosome probes accumulate in their respective organelles. The labeling concentration and hence the brightness can be varied over a broad range. The brightness of DNA, actin, and tubulin probes, on the other hand, cannot be varied to the same degree as their number of binding sites is limited.

The herein tested probes allowed simultaneous imaging of combinations of mitochondria, lysosomes, nucleus (DNA), F-actin, or microtubules. To access alternative targets for which no synthetic probes are available or for which the probes do not show a difference in fluorescence lifetime, the self-labeling protein (SLP) tag strategy can be employed. SLP tags, such as HaloTag7^24^ or SNAP-tag,^25^ react with fluorophores bearing a chloroalkane (CA) or benzylguanine (BG) ligand, respectively. Through fusion of the SLP tag to proteins of interest (POI), one can therefore localize cell-permeable fluorophores to different subcellular localizations. We previously demonstrated the use of SLP-tags in fluorescence lifetime multiplexing and characterized the average fluorescence lifetime of different HaloTag7 fusion proteins conjugated to MaP555-CA (2.4 ns) or MaP618-CA (3.1 ns).^17^ We hence performed a similar characterization for SNAP-tag localized to different subcellular localizations and labeled with MaP555-BG in living U-2 OS cells (e.g., histone 2B, Lamin B1, Tomm20 etc.; Supporting Table S4). SNAP-tag-MaP555 showed little variation in fluorescence lifetime averaging around 2.5 ns. The MaP555 substrates for SNAP (BG) and HaloTag (CA) should therefore be multiplexable with the SPY555 probes (Actin, DNA, and Tubulin) as they have a fluorescence lifetime difference of around 0.5 ns. To test this, we expressed HaloTag7-SNAP-tag as a fusion with the endoplasmic reticulum (ER) marker calreticulin (CalR)/KDEL in U-2 OS cells and labeled them with either MaP555-BG or MaP555-CA. When combined with SPY555-Actin or SPY555-DNA, two species could be separated on the basis of their fluorescence lifetime information (Supporting Figure S7). On the other hand, the fluorescence lifetimes of Rhodamine B and BioTracker Orange Lysosome are too similar to the corresponding SLP probes and therefore did not allow multiplexing. MaP618-CA (3.1 ns) can be used for multiplexing with SPY620-DNA but not SPY620-Actin (Supporting Figure S8).

We then combined fluorescence lifetime multiplexing with spectrally resolved detection. First, four probes were multiplexed in two spectral channels, each containing two probes separable in fluorescence lifetime (Figure 3A): LysoTracker-Green and MitoTracker-Green with two SPY555 probes (e.g., SPY555-DNA and SPY555-Tubulin). Living U-2 OS cells labeled with this combination were imaged in both the green and orange channel by FLIM. Separation of the two lifetime species in each spectral channel gave access to a four species image of mitochondria, lysosomes, the nucleus, and the microtubule network (Supporting Figure S9). Alternatively, SPY555-Tubulin could be replaced by SPY555-Actin revealing the F-actin network. Instead of combining the green and the orange channel, two red probes (SPY620-DNA and SPY620-Actin) can also be combined with the two probes in the green channel (LysoTracker-Green and MitoTracker-Green; Supporting Figure S10). More than four species can be accessed combining synthetic probes with SLP-tags. We hence expressed HaloTag7-SNAP-tag in the ER of U-2 OS cells and labeled them with LysoTracker-Green, MitoTracker-Green, MaP555-CA, SPY555-Actin, SPY700-DNA, and SiR700-Tubulin, allowing us to acquire six species’ images. Acquisition of all three channels and separation of the two lifetime species in each of them revealed lysosomes, mitochondria, the ER, F-actin, the nucleus, and microtubules (Supporting Figure S11). In addition, we were able to perform a time course experiment by repeated FLIM measurements allowing us to follow the movement of all six species over 6 min (Supporting Figure S12). Furthermore, by combining the SNAP-tag with two HaloTag variants (HaloTag7 and HaloTag11 (decreased fluorescence lifetime)),^17^ eight species could be imaged. We labeled cells expressing HaloTag7 in the ER, HaloTag11 in the Golgi, and SNAP-tag at the plasma membrane with LysoTracker-Green, MitoTracker-Green, MaP555-BG, SPY555-Actin, MaP618-CA, SPY700-DNA, and SiR700-Tubulin and acquired all four spectral channels (Figure 3B, Supporting Figure S13). As the labeling with multiple synthetic probes raises concerns of cell viability, we investigated whether the labeling influenced the percentage of living cells 20 h post labeling, and indeed no significant difference was found (Supporting Figure S14). The combination of fluorescence lifetime multiplexing with spectrally resolved detection hence allows to double the number of species imaged.

**Figure 3:**
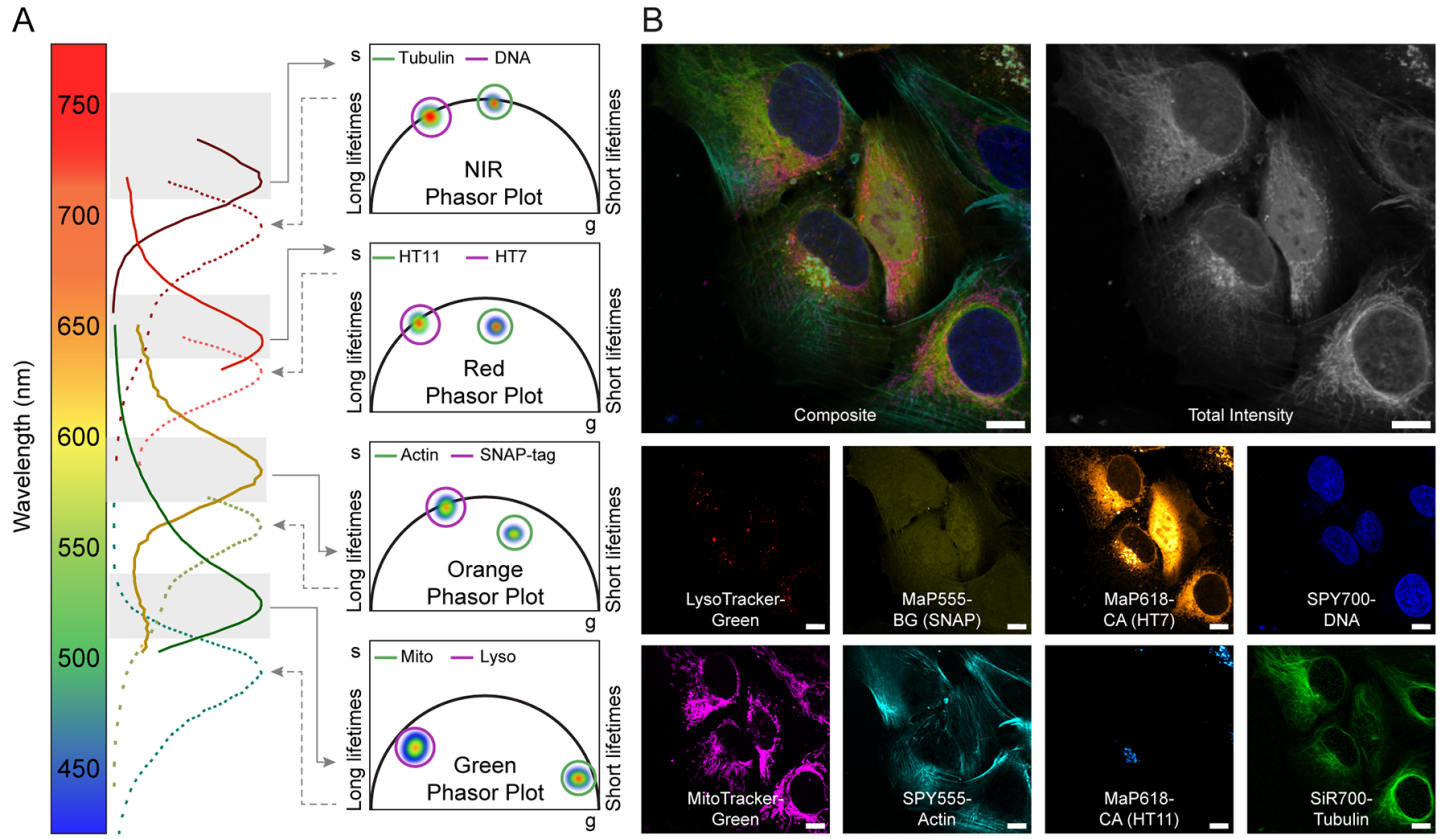
Combination of spectrally resolved detection and fluorescence lifetime multiplexing. (A) Schematic view of fluorescence lifetime multiplexing combined with spectrally resolved detection in four channels. (B) Fluorescence lifetime multiplexing of U-2 OS cells stably expressing a HaloTag7 fusion in the ER, HaloTag11 in the Golgi, and SNAP-tag at the plasma membrane. Cells were labeled with LysoTracker-Green, MitoTracker-Green, MaP555-BG, SPY555-Actin, MaP618-CA, SPY700-DNA, and SiR700-Tubulin. The composite, the total fluorescence intensity, and the eight individual separated species are given. Species separation was achieved using the phasor approach (positioning the cluster circles on the phasor plot at the position of the pure species). Scale bars, 10 μm.

In summary, the fluorescence lifetimes of 18 synthetic fluorescent probes were characterized, and eight pairwise combinations of probes suitable for fluorescence lifetime multiplexing were identified. In combination with spectrally resolved detection, these synthetic probes allow the doubling of the number of species that can be imaged, giving access to two and four species images and up to eight species when combined with SLP-tags. Fluorescence lifetime multiplexing via phasor analysis does not stop at pairs of probes but can technically also separate three species.^17^ This could open up the door to not only double but even triple the accessible species. However, this is currently limited as the phasors of suitable probes need to form a triangle, ideally an acute triangle (α, β, γ < 90°), in phasor space. None of the combinations of the 18 synthetic probes fulfilled these criteria. Separation of more than three species requires transformation into higher harmonics or the use of additional information.^9,10,26^ As more and more probes based on a variety of different fluorophores become available, we believe that it should be possible to expand the number of multiplexable species further and to access more species without genetic engineering. The use of synthetic probes targeting different biomolecules or cellular compartments is hence a straightforward strategy to generate fluorescence lifetime contrast and should facilitate the use of fluorescence lifetime multiplexing in living cells.

## Methods

### General Considerations

MaP555-BG, MaP555-CA, and MaP618-CA were prepared according to literature procedures^15^ by B. Réssy or D. Schmidt (MPI-MR). All other probes were purchased from commercial vendors (Supporting Table S1), or obtained from Spirochrome. Fluorophores were prepared as stock solutions in dry DMSO and diluted in imaging medium such that the final concentration of DMSO did not exceed 1% v/v.

### Confocal Microscopy

Confocal fluorescence microscopy was performed on a Leica SP8 FALCON microscope (Leica Microsystems) equipped with a Leica TCS SP8 X scanhead, a SuperK white light laser, Leica HyD SMD detectors, and a HC PL APO CS2 40 × 1.10 water objective. Emission was collected as indicated in Supporting Table S6. The microscope was equipped with a CO_2_ and temperature controllable incubator (Life Imaging Services, 37 °C).

Fluorescence excitation and emission spectra of synthetic probes were measured in living U-2 OS cells. For SNAP-tag and HaloTag7 probes, U-2 OS cells were transiently transfected with HaloTag7 or SNAP-tag (no localization marker). Settings can be found in the Supporting Methods.

### Fluorescence Lifetime Imaging Microscopy

FLIM was performed on a Leica SP8 FALCON microscope (as described above) at a pulse frequency of 80 MHz unless otherwise stated. Emission was collected as indicated in Supporting Table S6.

For determination of average intensity weighted fluorescence lifetimes of different probes cells were imaged, collecting 500 photons per pixel. The acquired images of cells were thresholded to remove the background signal from empty coverslip space. Mean fluorescence lifetimes were calculated in the LAS X software (Leica Microsystems) by fitting a mono-, bi-, or triexponential decay model (n-exponential reconvolution, unless otherwise stated) to the decay (χ^2^ < 1.2).

Crosstalk analysis was performed on the FLIM images acquired to determine the average intensity weighted fluorescence lifetimes. The contribution of a pure species into another species channel was determined by performing species separation via phasor analysis positioning the cluster circles on the phasor plot at the position of the pure species. The intensities of the two separated images were measured and the contribution of both of them to the total intensity calculated.

For determination of average intensity weighted fluorescence lifetimes on different subcellular targets, U-2 OS cells were transiently transfected with the SNAP-tag constructs and imaged, collecting 500 photons per pixel. The acquired images were processed as described above.

Structural images (species separation) were acquired as indicated in Supporting Table S6, and species separation was performed via phasor analysis positioning the cluster circles on the phasor plot at the position of the pure species (Leica Microsystems).^21,22,27^ No threshholding was applied.

For dynamic experiments, multiple images were acquired with a time delay of 2 min between the start of the first and the start of the second image.

**Data Availability.** Plasmids encoding SNAP-tag fusions were deposited on Addgene. Correspondence and reasonable requests for materials should be addressed to K.J.

## Supporting Information Available

Supporting phasor data; spectral characterization; two, four, six, and eight species images; chemical structures; lifetime values; plasmid information; microscopy data acquisition parameters (PDF)
