# Supplementary material for: Live-Cell Fluorescence Lifetime Multiplexing Using Synthetic Fluorescent Probes
Source: ACS Chem Biol. 2022 May 18;17(6):1321–7. doi: 10.1021/acschembio.2c00041 (PMC9207807; doi:10.1021/acschembio.2c00041)
Supplement: Supplementary file 1 — cb2c00041_si_001.pdf [file cb2c00041_si_001.pdf]

Supporting Information

Live-Cell Fluorescence Lifetime Multiplexing Using Synthetic Fluorescent Probes

Michelle S. Frei[1*], Birgit Koch[1], Julien Hiblot[1], Kai Johnsson[1-2*]

1 Department of Chemical Biology, Max Planck Institute for Medical Research, Jahnstrasse 29, 69120 Heidelberg, Germany

2 Institute of Chemical Sciences and Engineering (ISIC), École Polytechnique Fédérale de Lausanne (EPFL), 1015 Lausanne, Switzerland.

* Co-corresponding

michelle.s.frei@gmail.com

johnsson@mr.mpg.de

Table of Contents

Supporting Methods S3-5

Supporting Figures

Figure S1: Phasor plots of the 18 synthetic probes tested. S6

Figure S2: Spectral characterization of synthetic probes used for fluorescence lifetime multiplexing by confocal microscopy. S7

Figure S3: Exemplary crosstalk characterization of SPY555-DNA and SPY555-Tubulin in U-2 OS cells. S8

Figure S4: Live-cell fluorescence lifetime multiplexing using synthetic probes in different spectral regions. S9-10

Figure S5: Live-cell fluorescence lifetime multiplexing using synthetic probes in the orange spectral region. S11-12

Figure S6: Live-cell fluorescence lifetime multiplexing in different cell lines. S13

Figure S7: Multiplexing synthetic probes and self-labeling protein tags. S14-15

Figure S8: Multiplexing synthetic probes and HaloTag in the red channel. S16

Figure S9: Four species images combining fluorescence lifetime multiplexing in the green and orange spectral channel. S17

Figure S10: Four species images combining fluorescence lifetime multiplexing in the green and red spectral channel. S18

Figure S11: Six species images combining fluorescence lifetime multiplexing in the green, orange, and NIR spectral channel. S19

Figure S12: Dynamic six species images combining fluorescence lifetime multiplexing in the green, orange, and NIR spectral channel. S20

Figure S13: Eight species image combining fluorescence lifetime multiplexing in the green, orange, red, and NIR spectral channel. S21

Supporting Figure S14: Flow cytometry plots and statistical analysis of cell viability. S22

Supporting Tables

Table S1: Structures of all 18 synthetic probes tested. S23-26

Table S2: Comparison of intensity weighted fluorescence lifetimes (τ) of different synthetic probes in three different cell lines. S27
Table S3: Crosstalk analysis for all eight probe pairs. S28
Table S4: Comparison of intensity weighted fluorescence lifetimes (τ) of MaP555-BG on SNAPf-tag fused to different proteins of interest. S29
Table S5: Plasmids used and generated as well as the stable cell lines derived thereof. S30
Table S6: Fluorescence microscopy data acquisition parameters. S31-37
References S38-39

Supporting Methods

Plasmids: A pcDNA5/FRT/TO vector (ThermoFisher Scientific) was used for transient expression and stable cell line establishment in mammalian cells. HaloTag7-Pro30-SNAP-tag was fused to CalR/KDEL for ER localization. SNAPf-tag was fused to CEP41, H2B, TOMM20, NES, COX8, CalR/KDEL, β4Gal-T1, LAMP1, SKL, Lyn11, Ig-κ-/PDGFR, or Lifeact for expression in mammalian cells. Cloning was performed by Gibson assembly[1]. DNA was subsequently electroporated in *E. cloni*10G (Lucigene) and plated on agar Lysogenic Broth plates with 100 µg/mL ampicillin and incubated at 37 °C overnight. CEP41 (Addgene plasmid # 135446)[2], TOMM20 (Addgene plasmid # 135443)[2], H2B (Addgene plasmid # 135444)[2], COX8 (Addgene plasmid # 113916)[3], Ig-κ-/ -PDGFR[4], and SNAPf (Addgene plasmid # 167271)[5] were available in house and used as template plasmids. mCherry-LaminB1-10 was a gift from Michael Davidson (Addgene plasmid # 55069), pAAV_hsyn_NES-his-CAMPARI2-F391W-WPRE-SV40 was a gift from Eric Schreiter (Addgene plasmid # 101061)[6], pmTurquoise2-Golgi was a gift from Dorus Gadella (Addgene plasmid # 36205)[7], and LAMP1-mGFP was a gift from Esteban Dell'Angelica (Addgene plasmid # 34831)[8]. For more information see Supporting Table S5. Plasmids generated in this work have been deposited on Addgene and the accession codes are listed in Supporting Table S5.

Stable cell line establishment: The Flp-In™ T-REx™ System (ThermoFisher Scientific) was used to generate stable cell lines exhibiting inducible expression of CalR-HaloTag7-Pro30-SNAP-tag-KDEL or CalR-HaloTag7-KDEL-T2A-Lyn11-SNAPf. Briefly, pCDNA5-FRT-TO-GOI and pOG44 were co-transfected into the host cell line U-2 OS FlpIn TREx[9]. Homologous recombination between the FRT sites in pcDNA5-FRT-TO-GOI and the host cell chromosome, catalyzed by the Flp recombinase expressed from pOG44, produced the U-2 OS FlpIn TREx cells expressing stable GOI. Selection was performed using 100 µg mL^{-1} hygromycin B (ThermoFisher Scientific) and 15 µg mL^{-1} blasticidine (ThermoFisher Scientific).

Cell culture and transfection: U-2 OS (ATCC), HeLa (ATCC), HEK 293 (ATCC), U-2 OS Flp-In TREx CalR-HaloTag7-Pro30-SNAP-tag-KDEL, and U-2 OS Flp-In TREx CalR-HaloTag7-KDEL-T2A-Lyn11-SNAPf cells were cultured in high-glucose phenol red free DMEM (Life Technologies) medium supplemented with GlutaMAX (Life Technologies), sodium pyruvate (Life Technologies) and 10% FBS (Life Technologies) in a humidified 5% CO_2 incubator at 37 °C. Cells were split every 3–4 days or at confluency. Cell lines were regularly tested for mycoplasma contamination. Cells were seeded on 8 well glass bottom dishes (Ibidi) or 96 well plates (Eppendorf) three to one day before imaging. U-2 OS Flp-In TREx cells were induced using 100 µg ml^{-1} doxycycline (SigmaAldrich) for 24–48 h before imaging. Transient transfections were performed using Lipofectamine™ 2000 reagent (Life Technologies) according to the manufacturer's recommendations: the DNA (0.3 µg) was mixed with OptiMEM I (10 µL, Life Technologies) and Lipofectamine™ 2000 (0.75 µL) was mixed with OptiMEM I (10 µL, 8 well). The solutions were incubated for 5 min at room temperature, then mixed and incubated for an additional 20 min at room temperature. The prepared DNA-Lipofectamine complex was added to one of the wells in an 8 well glass bottom dish with cells at 50–70% confluency. After 12 h incubation in a humidified 5% CO_2 incubator at 37 °C the medium was changed to fresh medium. The cells were incubated under the same conditions for 24–48 h before imaging.

Labeling and sample preparation: Cells were labeled with the respective fluorophores:

- MitoTracker-Green: 200 nM, 30 min, 37 °C, one wash
- LysoTracker-Green: 75 nM, 30 min, 37 °C, one wash
- SPY555-Actin: 500 nM, 1 h , 37 °C, no wash
- SPY555-DNA: 1 µM, 1 h , 37 °C, no wash
- SPY555-Tubulin: 500 nM, 1 h , 37 °C, no wash
- MaP555-CA: 500 nM, 1 h , 37 °C, one wash
- MaP555-BG: 2 µM, 1 h , 37 °C, two washes
- MitoTracker-Orange: 500 nM, 30 min, 37 °C, one wash
- BioTracker 560 Orange Lysosomes: 10 µg of the fluorophore were diluted in 200 µL DMSO and a 1:1,000 dilution was used for labeling, 30 min, one wash
- Rodamine B: 1 µM, 30 min, 37 °C, one wash
- MitoTracker-Red: 500 nM, 30 min, 37 °C, one wash
- LysoTracker-Red: 75 nM, 30 min, 37 °C, one wash
- SPY620-Actin: 1 µM, 1 h , 37 °C, no wash
- SPY620-DNA: 500 nM, 1 h , 37 °C, no wash
- MaP618-CA: 500 nM, 1 h , 37 °C, one wash
- SiR-DNA: 500 nM, 1 h , 37 °C, no wash
- SiR-Tubulin: 500 nM, 1 h , 37 °C, no wash
- SiR-Actin: 500 nM, 1 h , 37 °C, no wash
- SPY700-DNA: 1 µM, 1 h , 37 °C, no wash
- SiR700-Tubulin: 500 nM, 1 h , 37 °C, no wash
- SiR700-Actin: 500 nM, 1 h , 37 °C, no wash

in phenol-red free DMEM medium supplemented with GlutaMAX, sodium pyruvate, and 10% FBS (all Life Technologies), washed with the same medium as indicated above. If no-wash and wash probes were used simultaneously, labeling with wash probes was performed first followed by labeling with no-wash probes. Imaging was performed in the same medium.

Fluorescence excitation and emission spectra: Fluorescence excitation and emission spectra of synthetic probes were measured in living U-2 OS cells by confocal microscopy. Acquisition settings were as follows:
Green excitation: excitation between 470-534 nm in 2 nm steps; collection at 555 – 600 nm.
Green emission: excitation at 470 nm and collection between 480–567 nm in 3 nm steps with a bandwidth of 10 nm.
Orange excitation: excitation between 475–575 nm in 2 nm steps; collection at 595–700 nm.
Orange emission: excitation at 520 nm and collection between 530–617 nm in 3 nm steps with a bandwidth of 10 nm.
Red excitation: excitation between 550–650 nm in 2 nm steps; collection at 670–780 nm.
Red emission: excitation at 600 nm and collection between 610–697 nm in 3 nm steps with a bandwidth of 10 nm.
Except LysoTracker-Red excitation: excitation between 500–600 nm in 2 nm steps; collection at 620–700 nm.
LysoTracker-Red emission: excitation at 540 nm and collection between 550–647 nm in 3 nm steps with a bandwidth of 10 nm.
NIR excitation: excitation between 630–670 nm in 2 nm steps; collection at 720–780 nm.

NIR emission: excitation at 670 nm and collection between 690–768 nm in 3 nm steps with a bandwidth of 10 nm.

Cell viability test

U-2 OS cells stably expressing CalR-HaloTag7-KDEL-T2A-Lyn11-SNAPf (induced with 100 µg ml^{-1} doxycycline) were transiently transfected with a plasmid expressing Golgi-HaloTag11 one day prior labeling. The cells were labeled with MaP618-CA (0.5 µM), MaP555-BG (2.0 µM) for 1 h whereby after 30 min LysoTracker-Green (75 nM) and MitoTracker-Green (200 nM) were added. Subsequently the cells were washed with growth medium and then labeled with SPY555-Actin (500 nM), SiR700-Tubulin (500 nM), and SPY700-DNA (1 µM) for ~20 h. The medium was collected from unlabeled and labeled cells. Additionally, the adherent cells were trypsinised and also added into the same tube to collect all dead and live cells. After spinning the cells at 300 g for 3 min, the cell pellet was resuspended in 2% FBS/PBS containing 1 µM SYTOX Blue dead cell stain (ThermoFisher). Technical triplicates were measured at the BD LSRFortessa™ X-20 Flow Cytometer (Becton, Dickinson and Company) using the software BD FACSDiva™. The following settings were used to detect the various channels:

**Channel**	**Laser**	**LP Filter**	**BP Filter**
SYTOX Blue	405nm		450/50
LysoTracker-Green/MitoTracker-Green	488nm	505LP	530/30
MaP555-BG/ SPY555-Actin	561nm	570LP	586/15
MaP618-CA/ SiR700-Tubulin/ SPY700-DNA	640nm	665LP	670/30

For flow cytometry analysis, 10,000 events were recorded and analysed using FlowJo 10.7.1.

Software and image processing: All images were processed in LASX and ImageJ/Fiji[10,11] unless otherwise stated. Excitation and emission spectra were visualized using OriginLab[12].

Supporting Figures

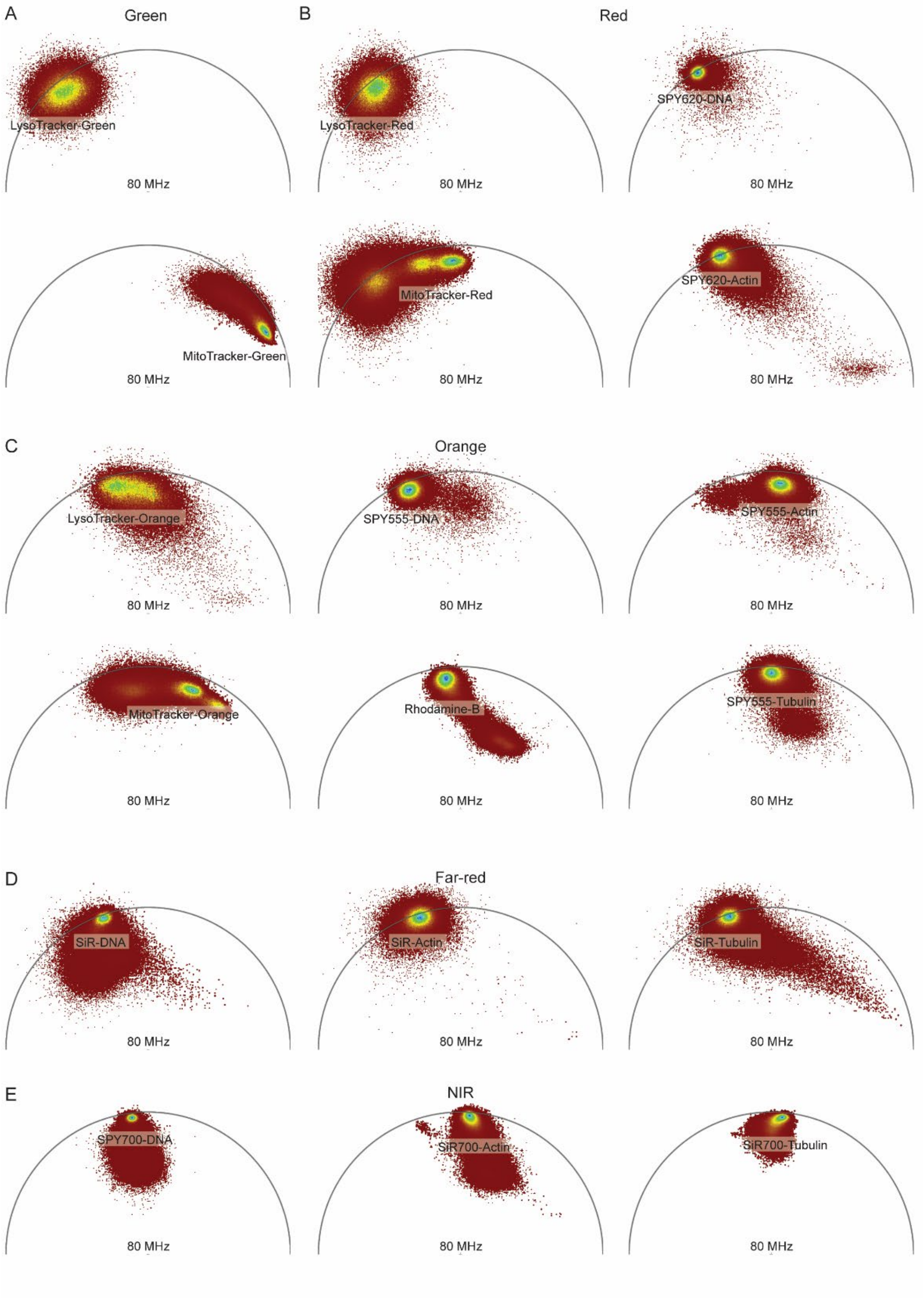

Supporting Figure S1: Phasor plots of the 18 synthetic probes tested. **A** Green spectral region. **B** Red spectral region. **C** Orange spectral region. **D** Far-red spectral region. **E** NIR spectral region.

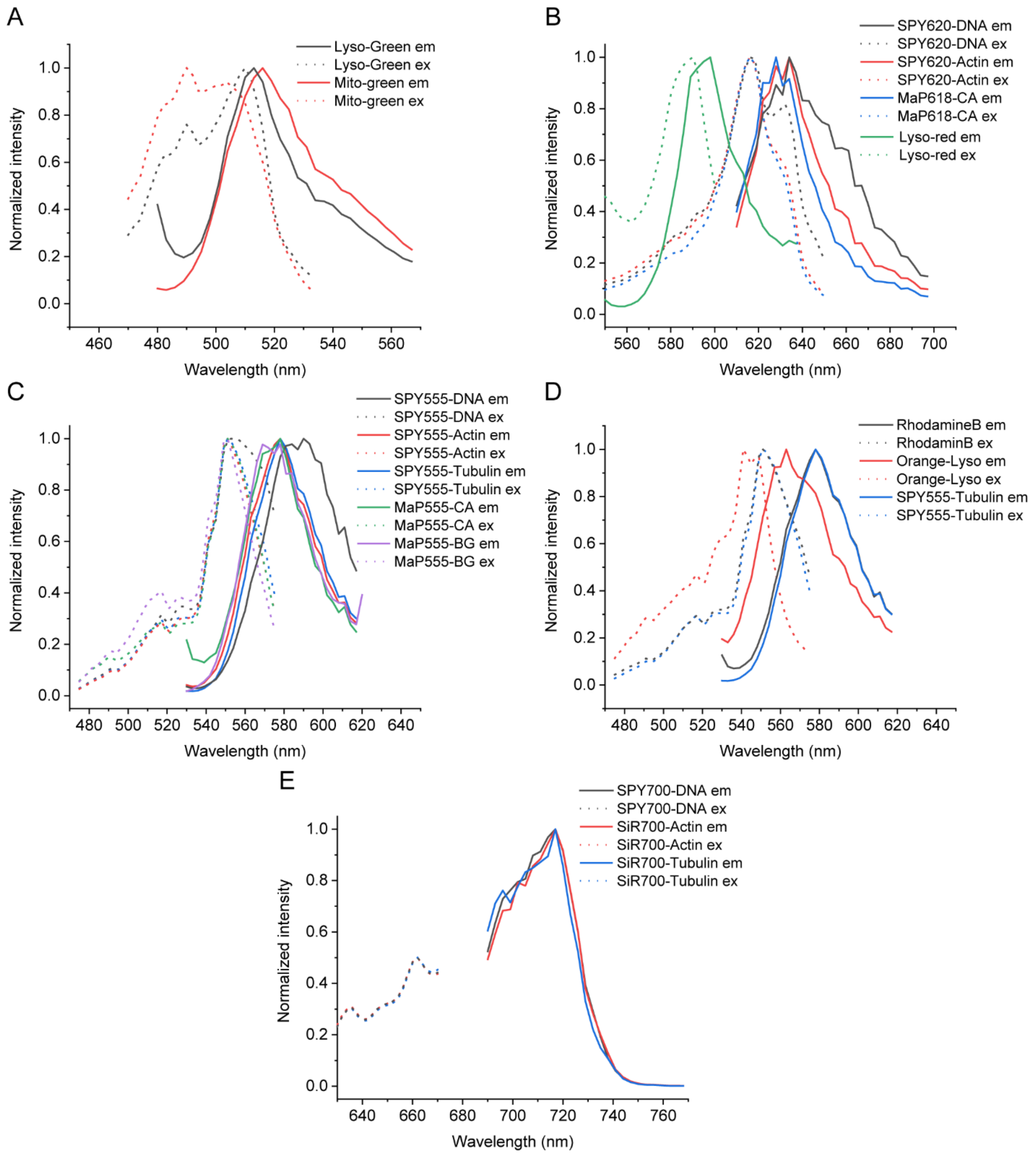

Supporting Figure S2: Spectral characterization of synthetic probes used for fluorescence lifetime multiplexing by confocal microscopy. **A-E** Normalized emission and excitation spectra of green probes (**A**), red probes (**B**), orange probes (**C**), orange probes (**D**), and NIR probes (**E**). For comparison of the orange probes the spectra of SPY555-Tubulin are given both in (**C**) and (**D**). The excitation maximum of the NIR probes (~680 nm) could not be measured as the laser only reached 670 nm. The spectra were normalized to 0.5 instead of 1.0.

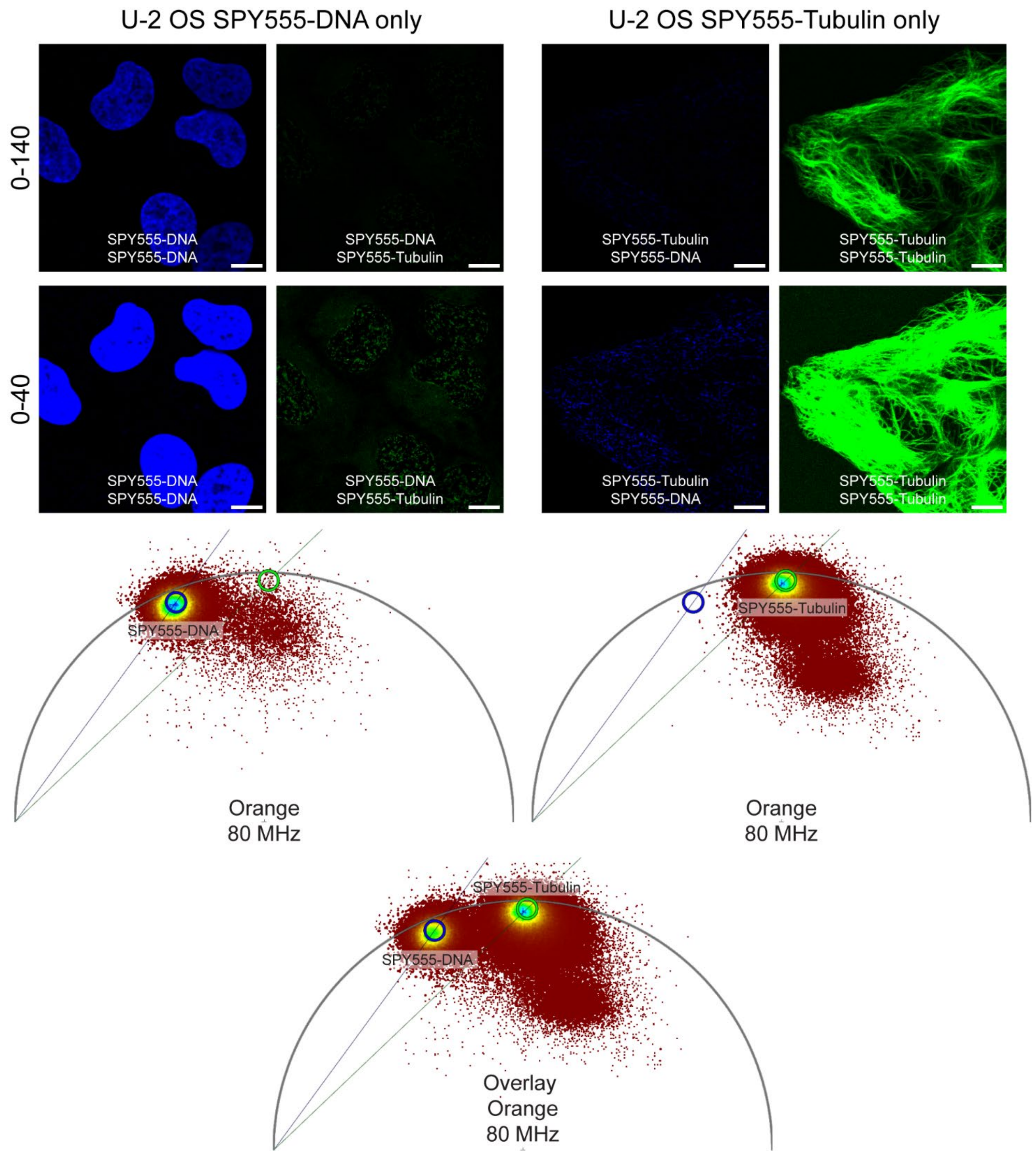

Supporting Figure S3: Exemplary crosstalk characterization of SPY555-DNA and SPY555-Tubulin in U-2 OS cells. Singly stained samples were used for the characterization and their phasor plots overlaid to define the position of the cluster circles. The two individual images were then separated resulting in the images of the pure species (SPY555-DNA – SPY555-DNA and SPY555-Tubulin-SPY55-Tubulin) as well as the images showing the crosstalk into the other lifetime component (SPY555-DNA-SPY555-Tubulin and SPY555-Tubulin-SPY555-DNA). These images were used to quantify the crosstalk (Supporting Table S3). To visualize the crosstalk and its relation to the pure species, the separated images were given in two different brightness scaling. Scale bars, 10 µm.

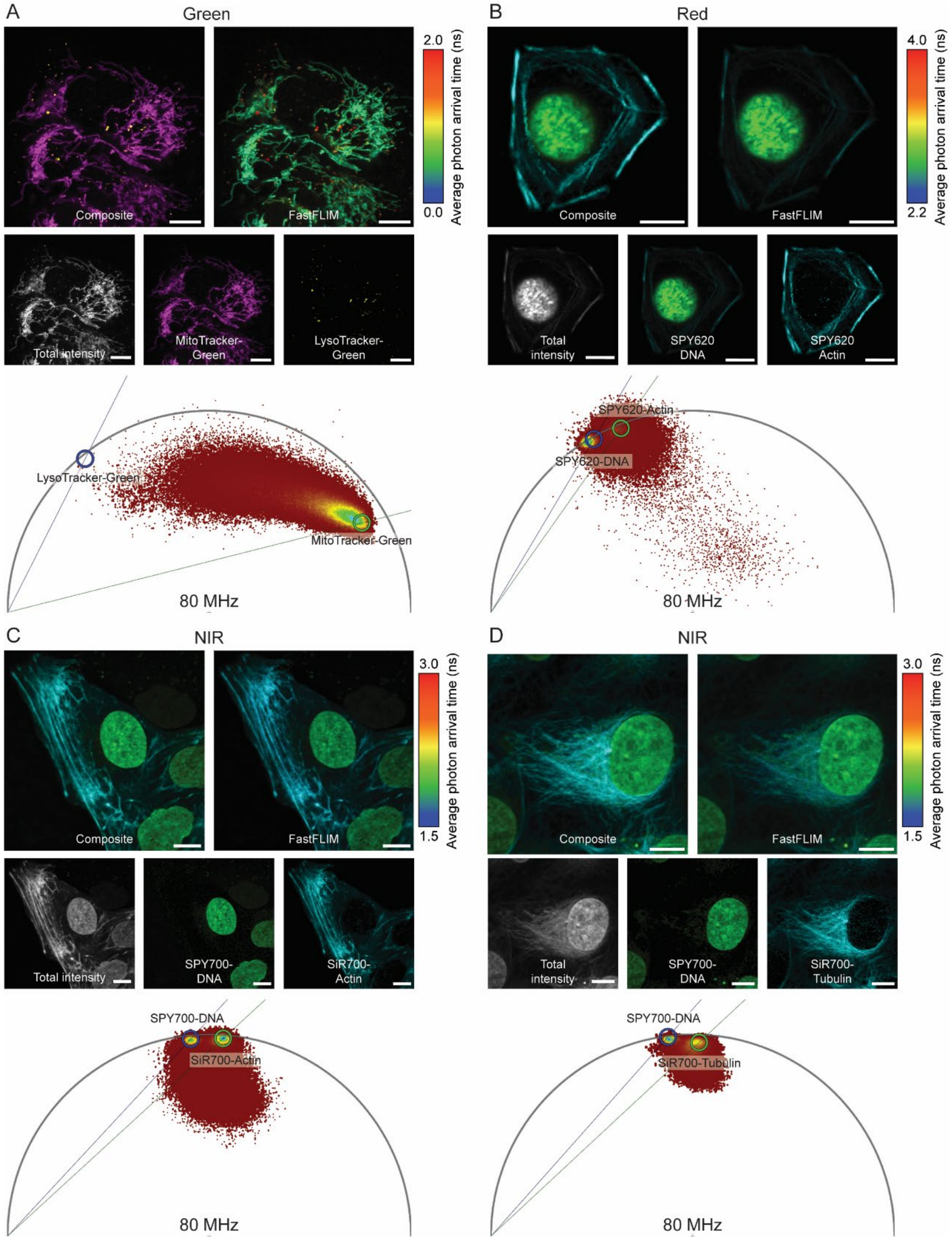

Supporting Figure S4: Live-cell fluorescence lifetime multiplexing using synthetic probes in different spectral regions. **A** Green spectral region labeling U-2 OS cells with MitoTracker-Green and LysoTracker-Green. **B** Red spectral region labeling U-2 OS cells with SPY620-DNA and SPY620-Actin. **C-D** NIR spectral region labeling U-2 OS cells with SPY700-DNA and SiR700-Actin (**C**) or SPY700-DNA and SiR700-Tubulin (**D**). The composite, the FastFLIM image with the respective color scale, the total fluorescence intensity, the two individual separated species as well as the corresponding wavelet-filtered phasor plot used for separation are given. Species separation was achieved

using the phasor approach by positioning the cluster circles on the phasor plot at the position of the pure species. Scale bars, 10 µm.

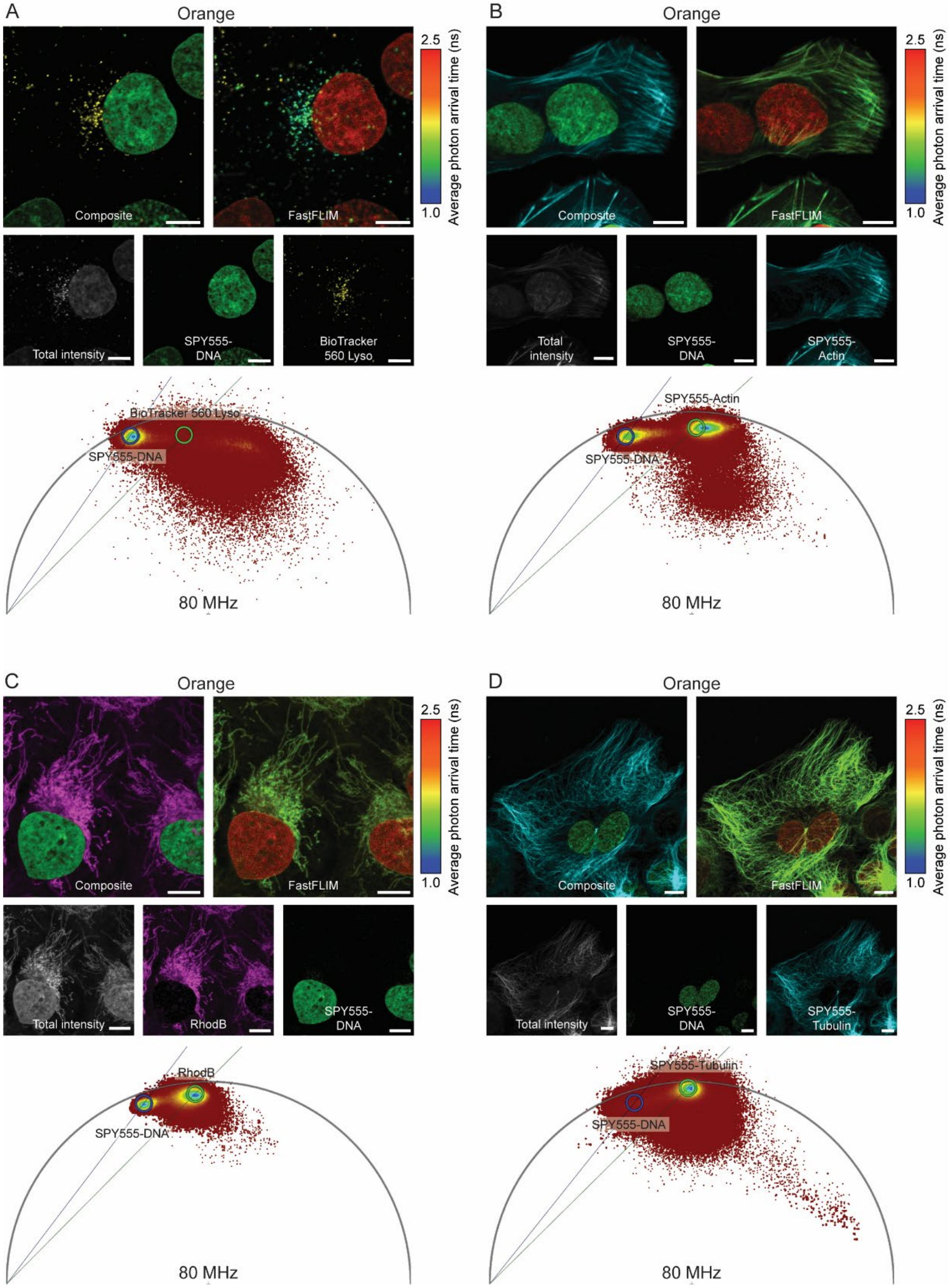

Supporting Figure S5: Live-cell fluorescence lifetime multiplexing using synthetic probes in the orange spectral region. **A-D** U-2 OS cells labeled with SPY555-DNA and BioTracker 560 Orange Lysosome (**A**), SPY555-DNA and SPY555-Actin (**B**), SPY555-DNA and RhodamineB (**C**), or SPY555-DNA and SPY555-Tubulin (**D**). The composite, the FastFLIM image with the respective color scale, the total fluorescence intensity, the two individual separated species as well as the corresponding wavelet-filtered phasor plot used for separation are given. Species separation

was achieved using the phasor approach by positioning the cluster circles on the phasor plot at the position of the pure species. Scale bars, 10 µm.

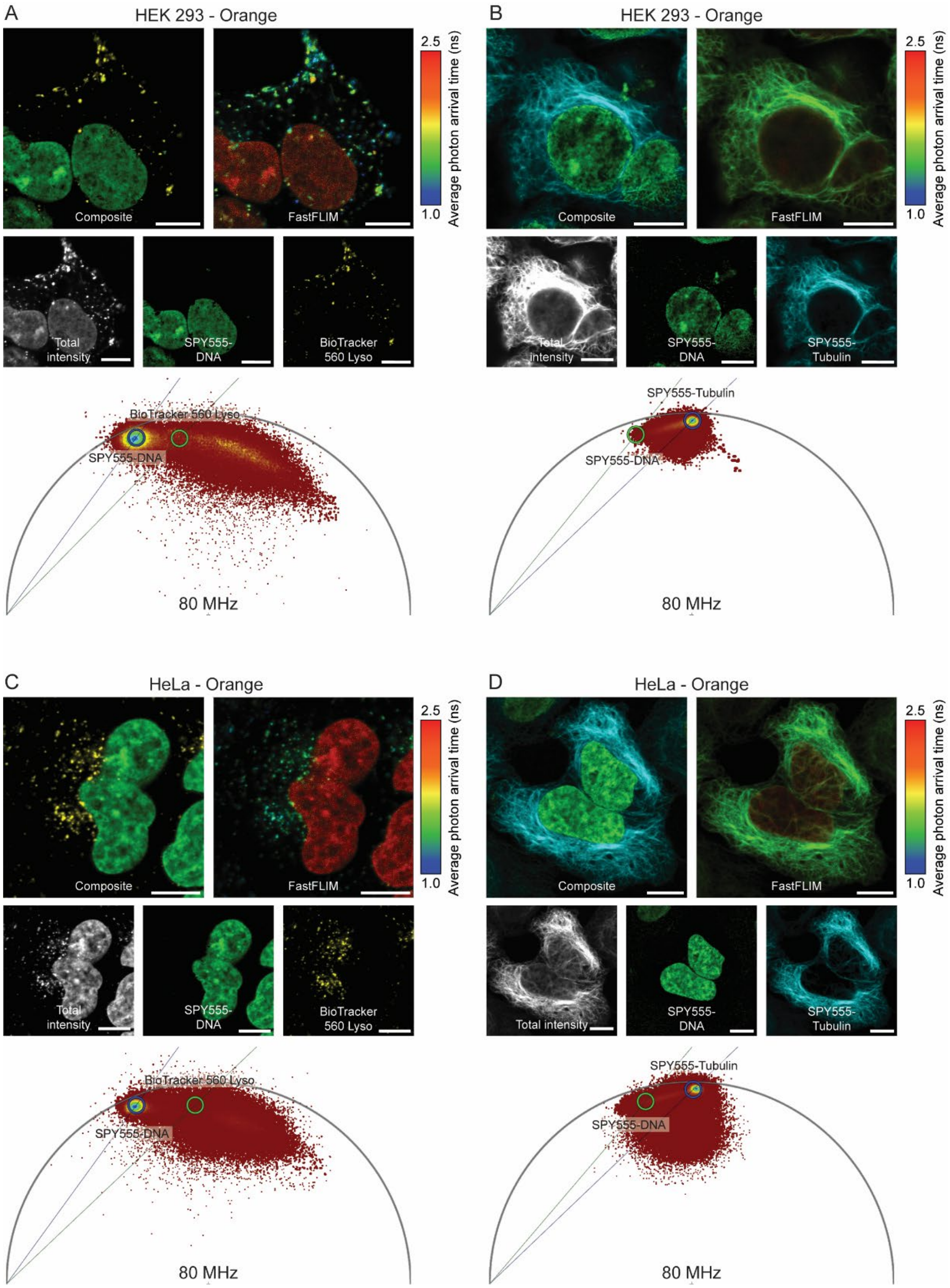

Supporting Figure S6: Live-cell fluorescence lifetime multiplexing in different cell lines. **A-D** HEK 293 (**A-B**) or HeLa cells (**C-D**) labeled with SPY555-DNA and BioTracker 560 Orange Lysosome (**A**, **C**) or SPY555-DNA and SPY555-Tubulin (**B**, **D**). The composite, the FastFLIM image with the respective color scale, the total fluorescence intensity, the two individual separated species as well as the corresponding wavelet-filtered phasor plot used for separation are given. Species separation was achieved using the phasor approach by positioning the cluster circles on the phasor plot at the position of the pure species. Scale bars, 10 µm.

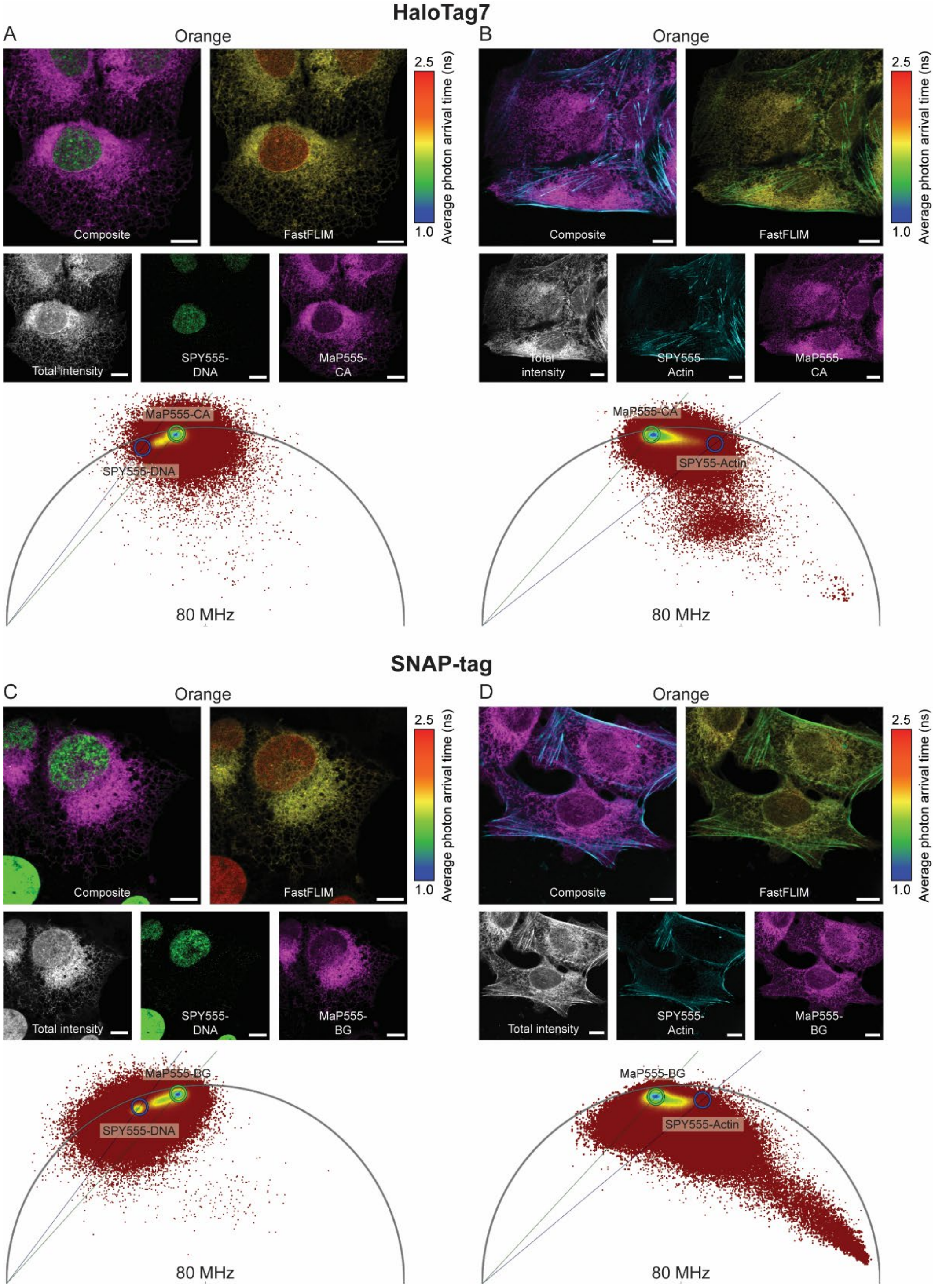

Supporting Figure S7: Multiplexing synthetic probes and self-labeling protein tags. **A-D** U-2 OS cells stably expressing the endoplasmic reticulum marker calreticulin (CalR) as a HaloTag7-SNAP-tag fusion additionally fused to a KDEL targeting peptide were labeled with MaP555-CA (**A**-**B**) or MaP555-BG (**C-D**) and SPY555-DNA (**A**, **C**) or SPY555-Actin (**B**, **D**). The composite, the FastFLIM image with the respective color scale, the total fluorescence intensity, the two individual separated species as well as the corresponding wavelet-filtered phasor plot used for

separation are given. Species separation was achieved using the phasor approach by positioning the cluster circles on the phasor plot at the position of the pure species. Scale bars, 10 µm.

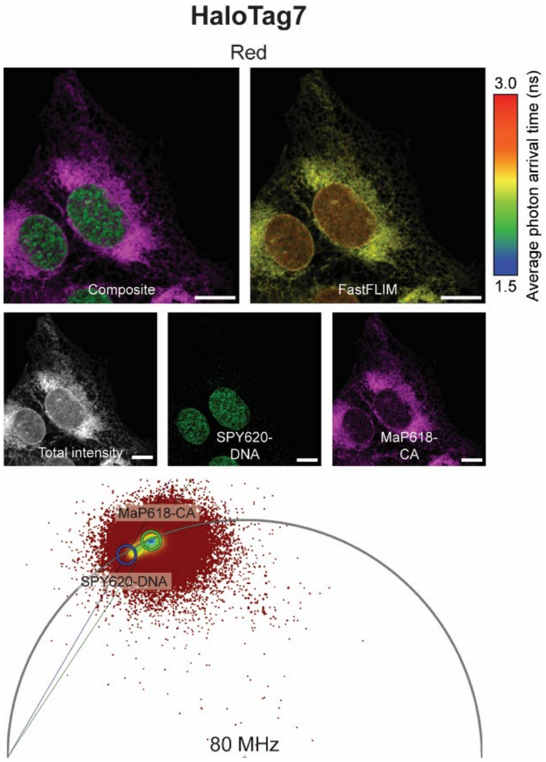

Supporting Figure S8: Multiplexing synthetic probes and HaloTag in the red channel. **A** U-2 OS cells stably expressing CalR-HaloTag7-SNAP-tag-KDEL were labeled with MaP618-CA and SPY620-DNA. The composite, the FastFLIM image with the respective color scale, the total fluorescence intensity, the two individual separated species as well as the corresponding wavelet-filtered phasor plot used for separation are given. Species separation was achieved using the phasor approach by positioning the cluster circles on the phasor plot at the position of the pure species. Scale bars, 10 µm.

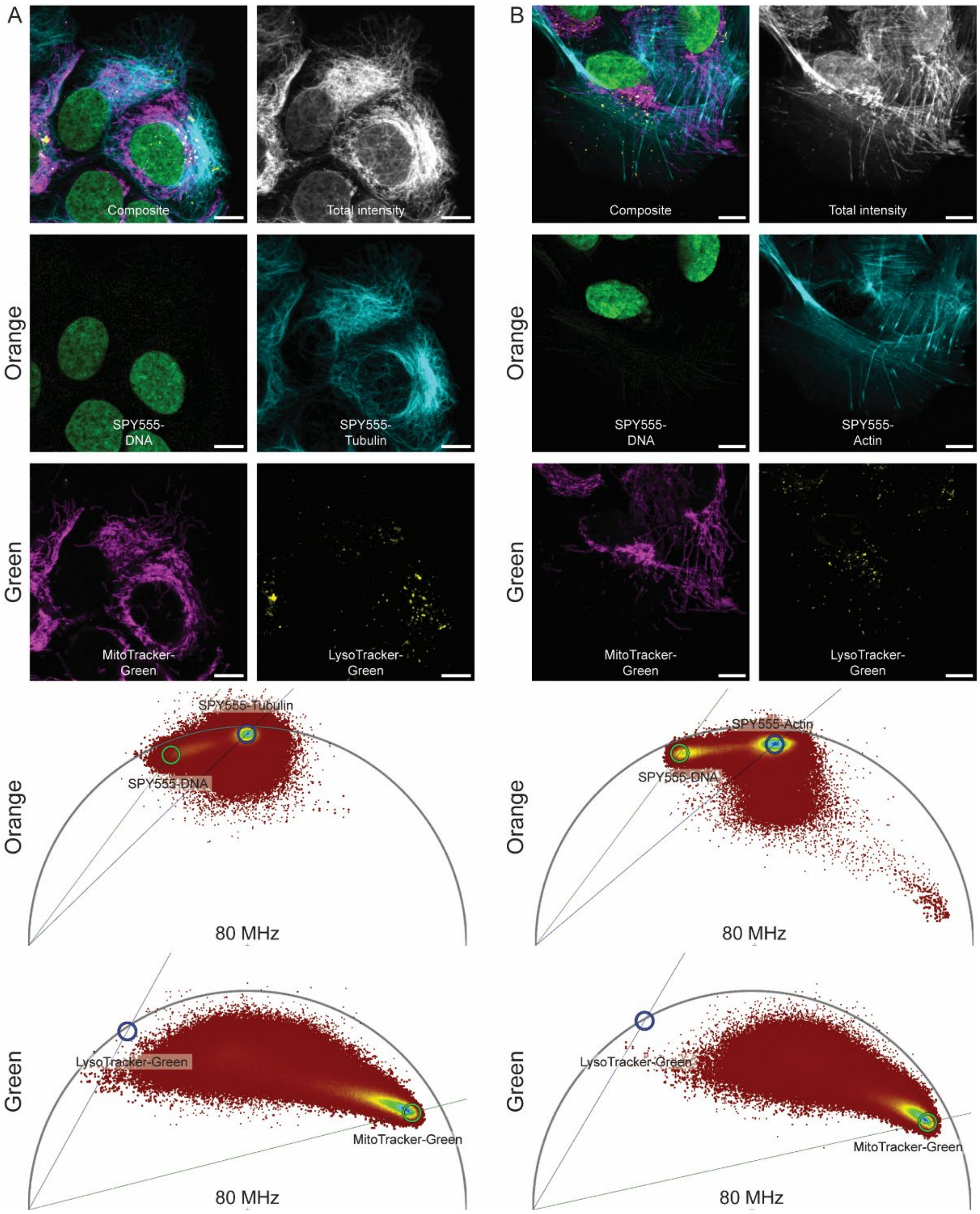

Supporting Figure S9: Four species images combining fluorescence lifetime multiplexing in the green and orange spectral channel. **A-B** U-2 OS cells were labeled with MitoTracker-Green, LysoTracker-Green, SPY555-DNA, and either SPY555-Tubulin (**A**) or SPY555-Actin (**B**) and imaged in the two spectral channels giving access to four species images. The composite, the total fluorescence intensity, the four individual separated species as well as the corresponding wavelet-filtered phasor plots used for separation are given. Species separation was achieved using the phasor approach by positioning the cluster circles on the phasor plot at the position of the pure species. Scale bars, 10 µm.

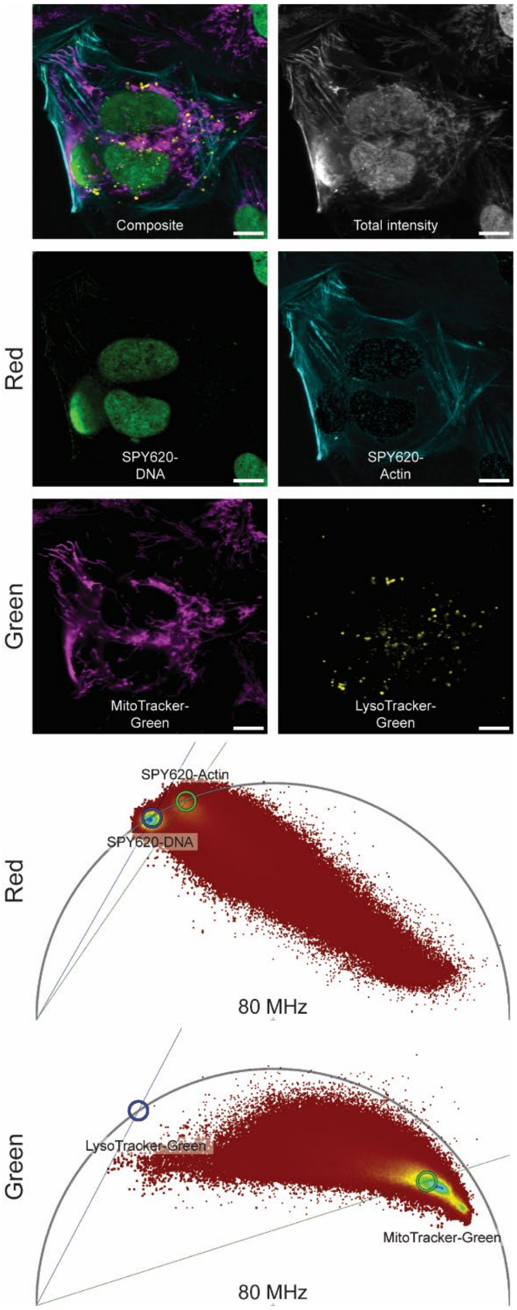

Supporting Figure S10: Four species images combining fluorescence lifetime multiplexing in the green and red spectral channel. U-2 OS cells were labeled with MitoTracker-Green, LysoTracker-Green, SPY620-DNA, and SPY620-Actin and imaged in the two spectral channels giving access to a four species image. The composite, the total fluorescence intensity, the four individual separated species as well as the corresponding wavelet-filtered phasor plots used for separation are given. Species separation was achieved using the phasor approach by positioning the cluster circles on the phasor plot at the position of the pure species. Scale bars, 10 µm.

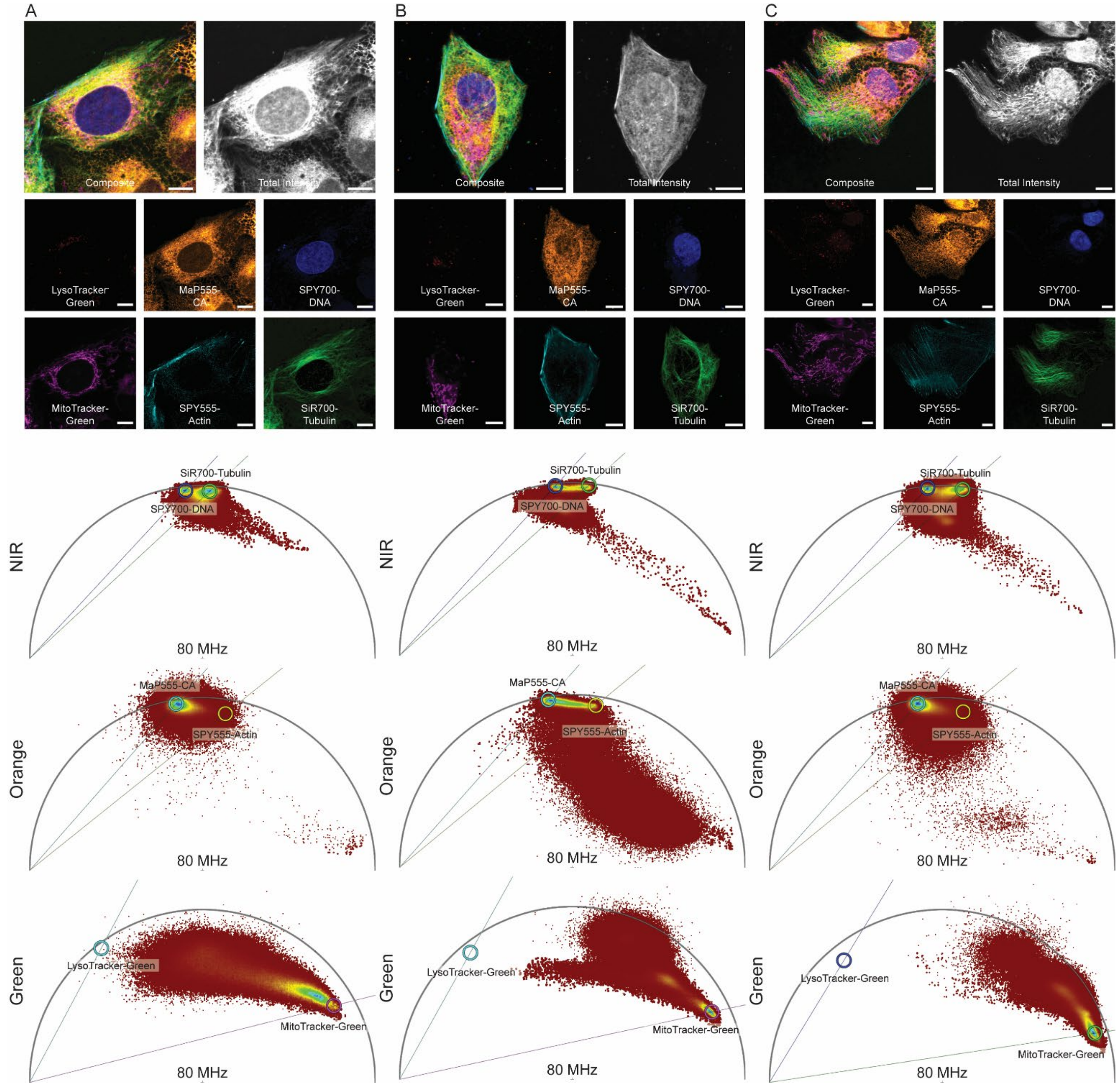

Supporting Figure S11: Six species images combining fluorescence lifetime multiplexing in the green, orange, and NIR spectral channel. **A-C** U-2 OS cells stably expressing CalR-HaloTag7-SNAP-tag-KDEL were labeled with MitoTracker-Green, LysoTracker-Green, MaP555-CA, SPY555-Actin, SPY700-DNA and SiR700-Tubulin and imaged in the three spectral channels giving access to six species images. The composite, the total fluorescence intensity, the six individual separated species as well as the corresponding wavelet-filtered phasor plots used for separation are given. Species separation was achieved using the phasor approach by positioning the cluster circles on the phasor plot at the position of the pure species. Scale bars, 10 µm.

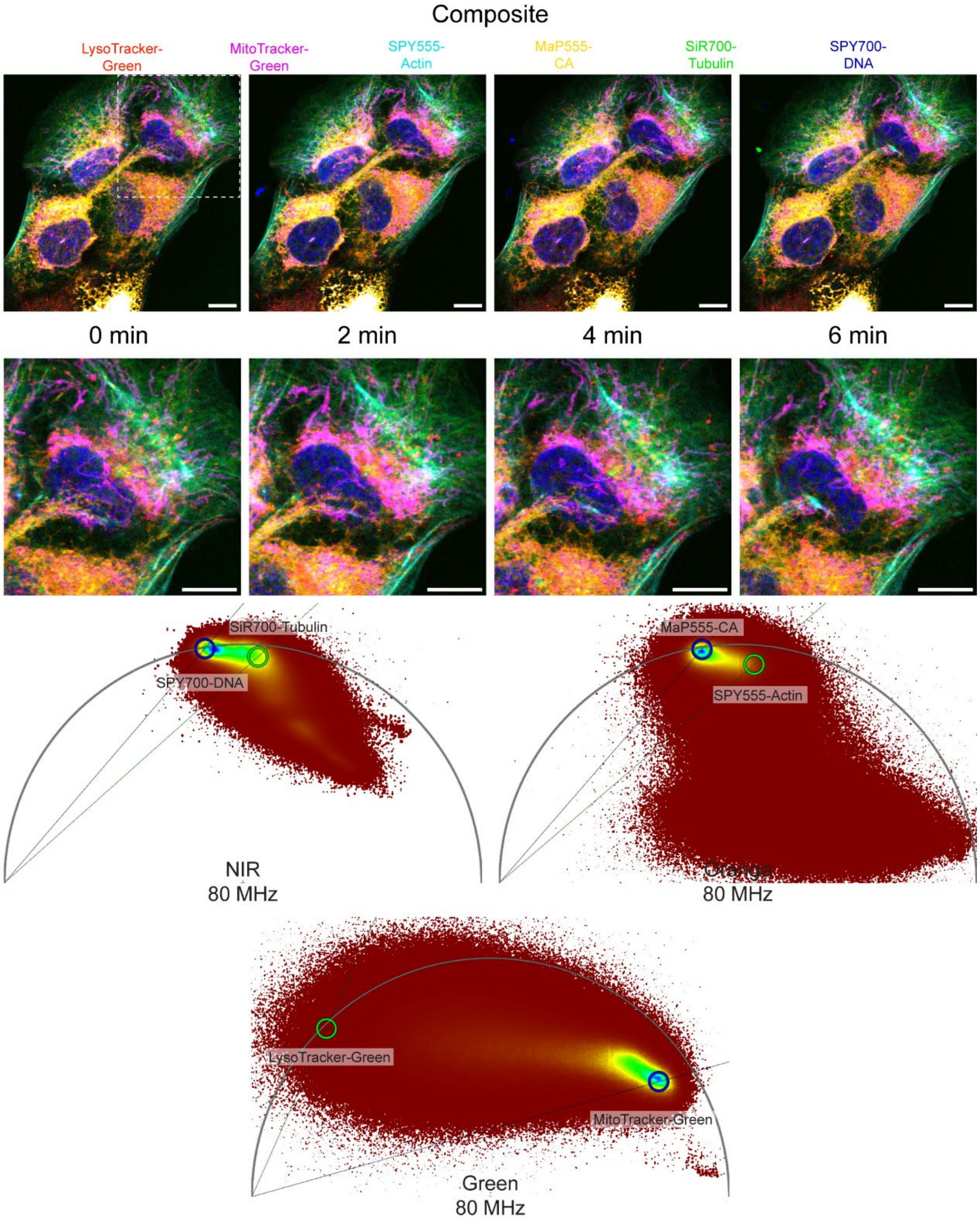

Supporting Figure S12: Dynamic six species images combining fluorescence lifetime multiplexing in the green, orange and NIR spectral channel. U-2 OS cells stably expressing CalR-HaloTag7-SNAP-tag-KDEL were labeled with MitoTracker-Green, LysoTracker-Green, MaP555-CA, SPY555-Actin, SPY700-DNA, and SiR700-Tubulin and repeatedly imaged in the three spectral channels giving access to six species images. Composites at the four different time points, a zoom as well as the corresponding summed wavelet-filtered phasor plots used for separation are given. Species separation was achieved using the phasor approach by positioning the cluster circles on the phasor plot at the position of the pure species. Scale bars, 10 µm.

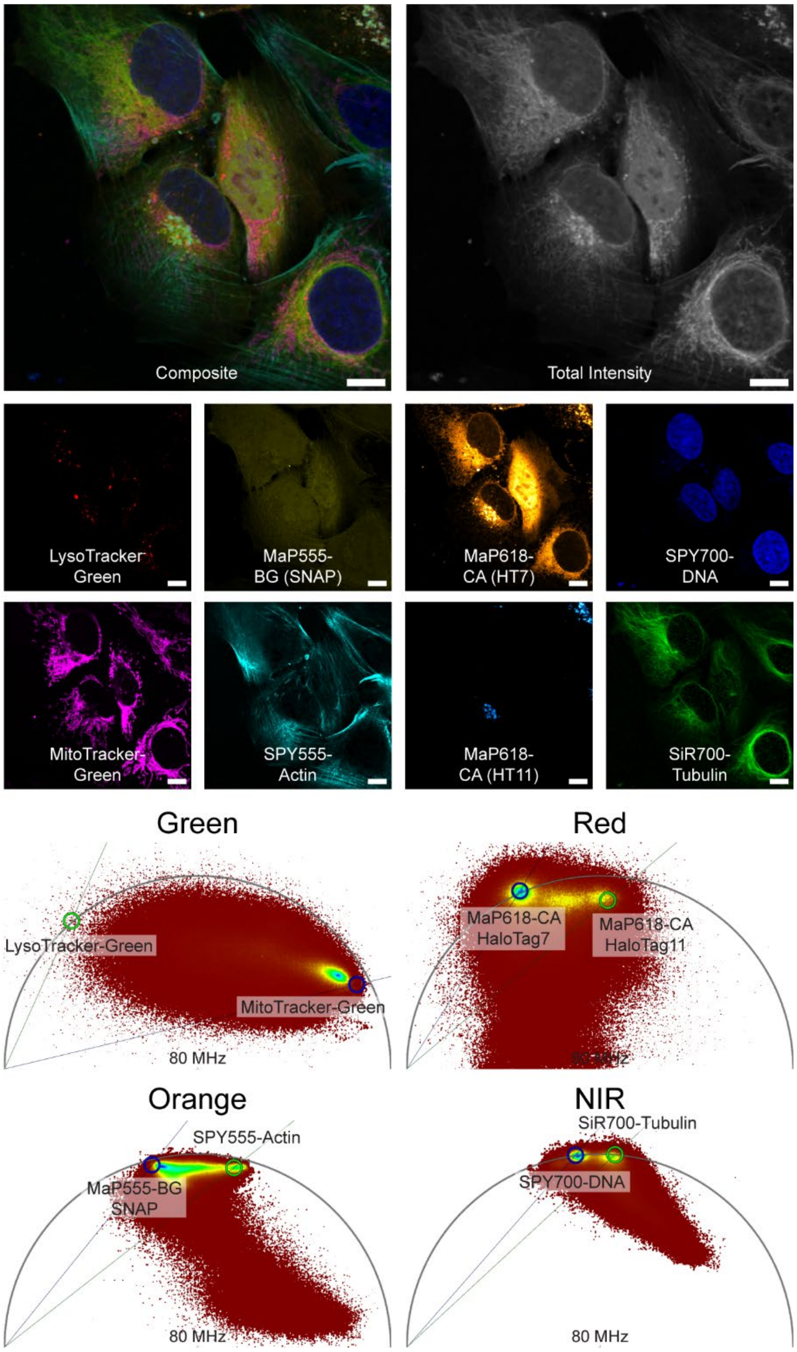

Supporting Figure S13: Eight species image combining fluorescence lifetime multiplexing in the green, orange, red, and NIR spectral channel. U-2 OS cells stably expressing CalR-HaloTag7-KDEL and Lyn11-SNAP-tag were transiently transfected with β-4-Gal-T1-HaloTag11 and labeled with MitoTracker-Green, LysoTracker-Green, MaP555-BG, SPY555-Actin, MaP618-CA, SPY700-DNA, and SiR700-Tubulin and imaged in the four spectral channels giving access to eight species images. The composite, the total fluorescence intensity, the eight individual separated species as well as the corresponding wavelet-filtered phasor plots used for separation are given. Species separation was achieved using the phasor approach by positioning the cluster circles on the phasor plot at the position of the pure species. Scale bars, 10 µm.

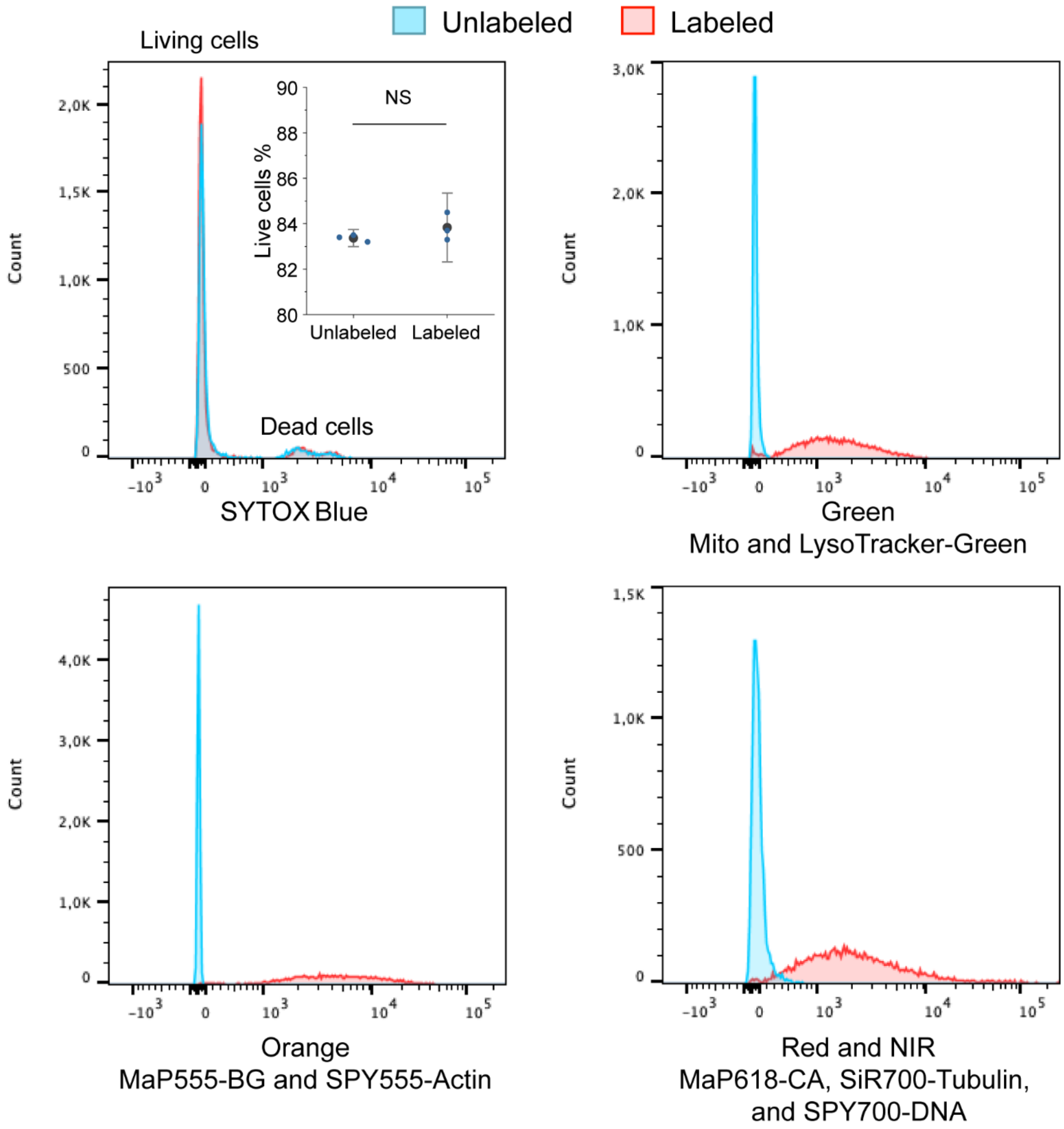

Supporting Figure S14: Flow cytometry plots and statistical analysis of cell viability. U-2 OS cells stably expressing CalR-HaloTag7-KDEL and Lyn11-SNAP-tag were transiently transfected with β-4-Gal-T1-HaloTag11 and labeled with MitoTracker-Green, LysoTracker-Green, MaP555-BG, SPY555-Actin, MaP618-CA, SPY700-DNA, and SiR700-Tubulin. After 20 h, all cells both dead (supernatant) and living (trypsination) were collected and labeled with SYTOX Blue. Flow cytometry analysis of four channels clearly showed labeled and unlabeled (Green, Orange, and the combined Red and NIR) as well as living and dead cells (SYTOX Blue). The difference in cell viability of labeled and unlabeled cells was not statistically significant (two sided t-test, $p = 0.27$, degrees of freedom = 4). The mean ± 95%CI and the three individual data points from technical replicates are given.

Supporting Tables

Supporting Table S1: Structures of all 18 synthetic probes tested. In addition, the vendor/reference and their excitation and emission maximum (as specified by the vendor) are given.

Name	Vendor/ Reference	Ex/Em [nm]	Structure
**MitoTracker-Green**	ThermoFisher Scientific	490/516	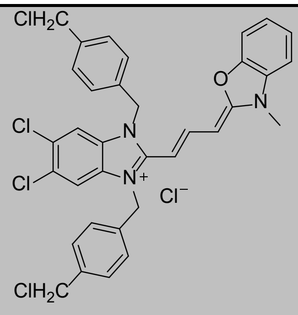 MitoTracker™-Green
**LysoTracker-Green**	ThermoFisher Scientific	504/511	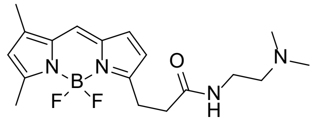 LysoTracker™-Green
**SPY555-Actin**	Spirochrome	555/580	Unpublished
**SPY555-DNA**	Spirochrome	555/580	Unpublished
**SPY555-Tubulin**	Spirochrome	555/580	Unpublished
**MaP555-CA**	[13]	558/578	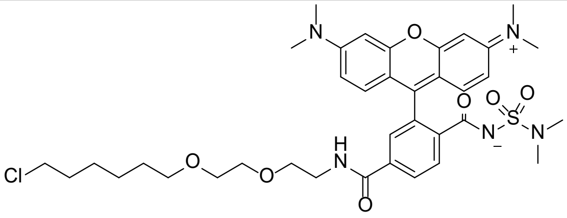 MaP555-CA
**MaP555-BG**	[13]	556/576	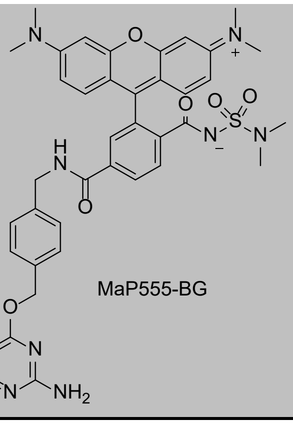 MaP555-BG

**MitoTracker-Orange**	ThermoFisher Scientific	554/576	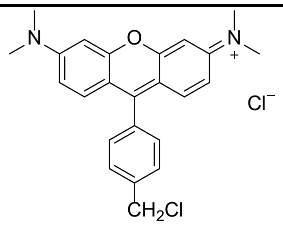 MitoTracker^{TM}-Orange
**Rhodamine B**	TCI	-	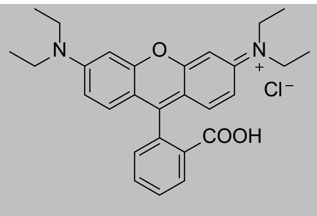 Rhodamine B
**BioTracker 560 Orange Lysosomes**	Merck	532/560	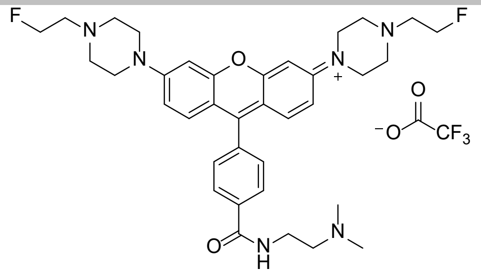 BioTracker 560 Orange Lysosome
**MitoTracker-Red**	ThermoFisher Scientific	579/599	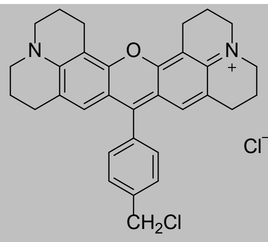 MitoTracker^{TM}-Red
**LysoTracker-Red**	ThermoFisher Scientific	577/590	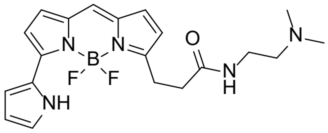 LysoTracker^{TM}-Red
**SPY620-Actin**	Spirochrome	619/636	Unpublished
**SPY620-DNA**	Spirochrome	618/636	Unpublished
**MaP618-CA**	[13]	618/635	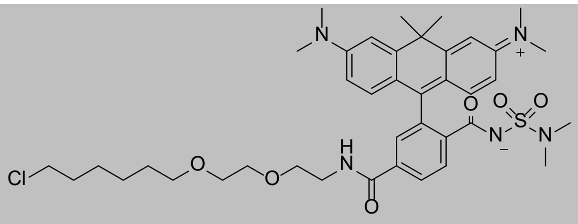 MaP618-CA

**SiR-DNA**	Spirochrome[14]	652/674	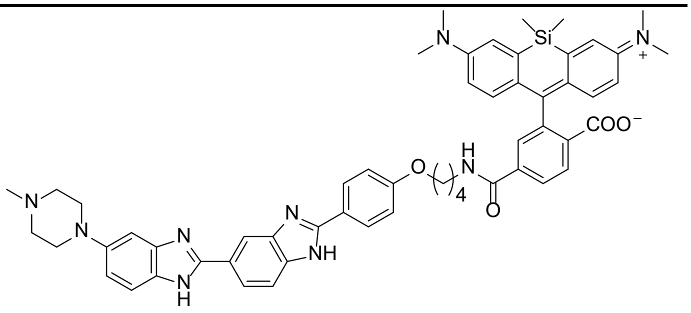 SiR-DNA
**SiR-Tubulin**	Spirochrome[15]	652/674	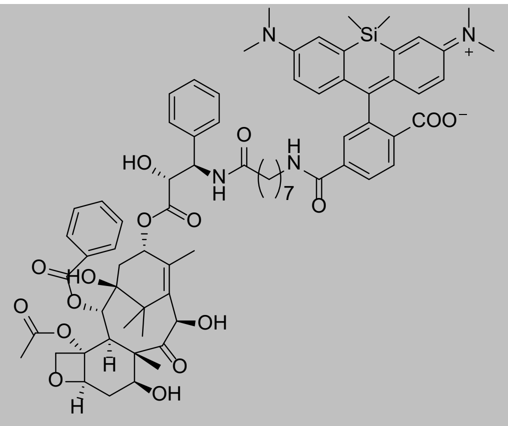 SiR-Tubulin
**SiR-Actin**	Spirochrome[15]	652/674	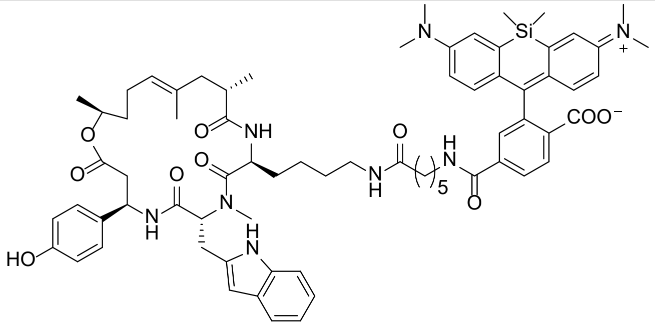 SiR-Actin
**SPY700-DNA**	Spirochrome	689/716	Unpublished
**SiR700-Tubulin**	Spirochrome[16]	689/716	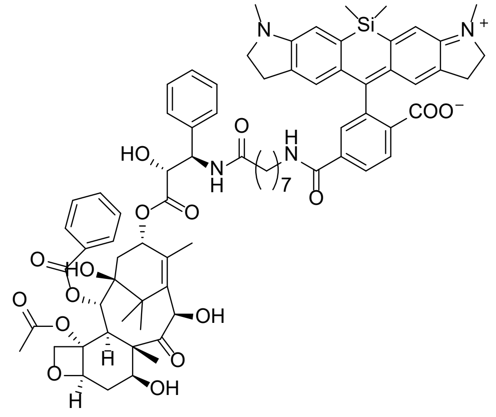 SiR700-Tubulin

**SiR700-Actin**	Spirochrome[16]	689/716	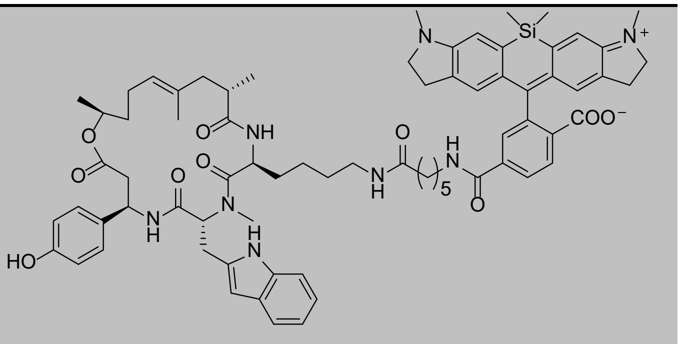 SiR700-Actin

Supporting Table S2: Comparison of intensity weighted fluorescence lifetimes (τ) of different synthetic probes in three different cell lines. Fluorescence lifetimes were measured by FLIM (mean ± SEM., *N* = 4 field of views (FOVs) from 2 biological replicates, average *N* = 12 FOVs from 6 biological replicates).[2] bi-exponential fit, [3] tri-exponential fit, * tail fit (all others n-exponential reconvolution fit).

	Fluorophore	U-2 OS [ns]		HeLa [ns]		HEK 293 [ns]		Average [ns]	
		τ	SEM	τ	SEM	τ	SEM	τ	SEM
Green	MitoTracker-Green	0.96[3*]	0.03	0.88[3*]	0.03	0.96[3*]	0.02	0.93	0.02
	LysoTracker-Green	3.92[2]	0.06	3.78[2]	0.05	4.24[2]	0.01	3.98	0.06
Orange	Rhodamine B	2.26[3]	0.03	2.12[3]	0.01	2.30[3]	0.02	2.23	0.03
	BioTracker Orange Lysosome	2.30[2]	0.07	1.80[3]	0.13	2.04[2]	0.05	2.05	0.08
	SPY555-Actin	1.79[2]	0.09	1.91[2]	0.02	1.88[2]	0.03	1.86	0.03
	SPY555-DNA	3.00[2]	0.02	3.01[2]	0.04	3.01[2]	0.01	3.01	0.01
	SPY555-Tubulin	2.00[2]	0.01	1.99[2]	0.01	2.02[2]	0.01	2.00	0.01
Red	LysoTracker-Red	3.88[2]	0.07	3.57[2]	0.09	3.63[2]	0.06	3.70	0.06
	SPY620-Actin	2.93[2]	0.01	2.94[2]	0.01	2.88[2]	0.03	2.91	0.01
	SPY620-DNA	3.62[2]	0.01	3.59[2]	0.01	3.61[2]	0.01	3.60	0.01
NIR	SiR700-Actin	1.90[2]	0.01	1.88[2]	0.01	1.93[2]	0.01	1.90	0.01
	SPY700-DNA	2.28[2]	0.01	2.26[2]	0.01	2.30[2]	0.01	2.28	0.01
	SiR700-Tubulin	1.90[3]	0.01	1.90[3]	0.01	2.02[3]	0.01	1.94	0.02

Supporting Table S3: Crosstalk analysis for all eight probe pairs. Images of singly stained U-2 OS cells were separated for the two species under question and the resulting images used to calculate crosstalk (mean ± SEM, N = 4 field of views (FOVs) from 2 biological replicates).

	**Fluorophore**	**Pure**		**Crosstalk**	
		%	SEM	%	SEM
**Green**	**MitoTracker-Green**	96.6	0.5	3.4	0.5
	**LysoTracker-Green**	97.0	0.6	3.0	0.6
**Orange**	**Rhodamine B**	95.5	0.3	4.5	0.3
	**SPY555-DNA**	90.7	1.0	9.3	1.0
**Orange**	**BioTracker Orange Lysosome**	88.3	4.7	11.7	4.7
	**SPY555-DNA**	85.8	1.6	14.2	1.6
**Orange**	**SPY555-Actin**	94.0	0.5	6.0	0.5
	**SPY555-DNA**	94.5	0.7	5.5	0.7
**Orange**	**SPY555-Tubulin**	96.0	0.7	4.0	0.7
	**SPY555-DNA**	93.8	0.7	6.2	0.7
**Red**	**SPY620-Actin**	96.8	0.5	3.2	0.5
	**SPY620-DNA**	91.6	0.7	8.4	0.7
**NIR**	**SiR700-Actin**	91.7	1.3	8.3	1.3
	**SPY700-DNA**	95.2	1.3	4.8	1.3
**NIR**	**SiR700-Tubulin**	90.8	1.1	9.2	1.1
	**SPY700-DNA**	86.6	1.9	13.4	1.9

Supporting Table S4: Comparison of intensity weighted fluorescence lifetimes (τ) of MaP555-BG on SNAPf-tag fused to different proteins of interest. H2B: nucleus, labeling histone 2B, LMNB1: nuclear lamina, labeling Lamin B1, Cytosol: untargeted (no fusion), NES: cytosol, fusion with a nuclear export signal, Tomm20: outer mitochondrial membrane, labeling the membrane receptor Tomm20, COX8: inner mitochondrial membrane, labeling the cytochrome c oxidase subunit 8, CalR-KDEL: endoplasmic reticulum, labeling calreticulin and additionally fused to a KDEL targeting peptide, β-4-Gal-T1: Golgi Apparatus, labeling beta-1,4-galactosidase, LAMP1: lysosomes, labeling lysosome-associated membrane glycoprotein 1, SKL: peroxisomes, targeted via a peroxisomal targeting signal, Lyn11: inner leaflet of the plasma membrane, labeling tyrosine protein kinase Lyn11, Ig-κ-PDGF: outer plasma membrane, labeling platelet-derived growth factor receptor and additionally fused to an immunoglobulin kappa light chain leader sequence, CEP41: microtubules, labeling the microtubule-binding protein CEP41, lifeact: filamentous actin (F-actin), labeling the actin-binding peptide lifeact. Fluorescence lifetime was measured by FLIM (mean ± SEM, *N* = 4 FOVs from 2 biological replicates). Unless otherwise stated all mono-exponential fits. [2] bi-exponential. Data for HaloTag7 from reference[17].

**Fluorophore**	**SNAP-tag [ns]**		**HaloTag7 [ns]**	
	τ	SEM	τ	SEM
**H2B**	2.55[2]	0.03	2.41	0.01
**LMNB1**	2.57[2]	0.04	2.38	0.00
**Cytosol**	2.49[2]	0.01	2.33	0.01
**NES**	2.57[2]	0.02	2.41[2]	0.01
**Tomm20**	2.51[2]	0.02	2.38	0.00
**COX8**	2.46[2]	0.04	2.40[2]	0.02
**CalR-KDEL**	2.52[2]	0.03	2.36	0.00
**β4Gal-T1**	2.58[2]	0.14	2.35	0.03
**LAMP1**	2.62[2]	0.02	2.38	0.00
**SKL**	2.40[2]	0.05	2.37	0.01
**Lyn11**	2.37[2]	0.05	2.37[2]	0.01
**Ig-κ-PDGF**	2.34[2]	0.03	2.31[2]	0.01
**CEP41**	2.40[2]	0.06	2.51[2]	0.02
**Lifeact**	2.48[2]	0.03	2.36[2]	0.01
**Average**	2.49	0.06	2.38	0.01

Supporting Table S5: Plasmids used and generated as well as the stable cell line derived thereof.

Name	Addgene#	Plasmid	Gene	Localization tag Addgene#	Stable cell lines
**pCDNA5/FRT/TO_H2B-SNAPf-tag**	181998	pCDNA5/FRT/TO	H2B-SNAPf-tag	135444[2]	-
**pCDNA5/FRT/TO_SNAPf-tag-LMNB1**	181999	pCDNA5/FRT/TO	SNAPf-tag-LMNB1	55069	-
**pCDNA5/FRT/TO_SNAPf-tag**	182000	pCDNA5/FRT/TO	SNAPf-tag	167271[5]	-
**pCDNA5/FRT/TO_NES-SNAPf-tag**	182001	pCDNA5/FRT/TO	NES-SNAPf-tag	101061[6]	-
**pCDNA5/FRT/TO_TOMM20-SNAPf-tag**	182002	pCDNA5/FRT/TO	TOMM20-SNAPf-tag	135443[2]	-
**pCDNA5/FRT/TO_COX8-SNAPf-tag**	182003	pCDNA5/FRT/TO	COX8-SNAPf-tag	113916[3]	-
**pCDNA5/FRT/TO_CalR-SNAPf-tag -KDEL**	182004	pCDNA5/FRT/TO	CalR-SNAPf-tag-KDEL	synthetic	-
**pCDNA5/FRT/TO_β4Gal-T1-SNAPf-tag**	182005	pCDNA5/FRT/TO	β4Gal-T1-SNAPf-tag	36205[7]	-
**pCDNA5/FRT/TO_LAMP1-SNAPf-tag**	182006	pCDNA5/FRT/TO	LAMP1-SNAPf-tag	34831[8]	-
**pCDNA5/FRT/TO_SNAPf-tag-SKL**	182007	pCDNA5/FRT/TO	SNAPf-tag-SKL	synthetic	-
**pCDNA5/FRT/TO_Lyn11-SNAPf-tag**	182008	pCDNA5/FRT/TO	Lyn11-SNAPf-tag	synthetic	-
**pCDNA5/FRT/TO_Ig-κ-SNAPf-tag-PDGFR**	182009	pCDNA5/FRT/TO	Ig-κ-SNAPf-tag-PDGFR	[4]	-
**pCDNA5/FRT/TO_CEP41-SNAPf**	182010	pCDNA5/FRT/TO	CEP41-SNAPf	135446[2]	-
**pCDNA5/FRT/TO_Lifeact-SNAPf-tag**	182011	pCDNA5/FRT/TO	Lifeact-SNAPf-tag	aynthetic	-
**pCDNA5/FRT/TO_CalR-HaloTag7-P30-SNAP-tag-KDEL**	182012	pCDNA5/FRT/TO	CalR-SNAPf-tag-KDEL	synthetic	U-2 OS Flp-In TREx
**pCDNA5/FRT/TO_CalR-HaloTag7-KDEL-T2A-Lyn11-SNAPf**	-	pCDNA5/FRT/TO	CalR-HaloTag7-KDEL and Lyn11-SNAPf	synthetic	U-2 OS Flp-In TREx
**pCDNA5/FRT/TO_β4Gal-T1-HaloTag11**[17]	-	pCDNA5/FRT/TO	β4Gal-T1-HaloTag11	36205[7]	-

Supporting Table S6: Fluorescence microscopy data acquisition parameters. *See methods for details.

Image	Probe	Microscope	Excitation [nm]	Pixel dwell time [µs]	Pinhole [Airy Units]	Objective	Pixel size [nm]	Size [pixels]	Emission [nm]	Comment
**Fig. 1C**	MitoTracker-Green	SP8-FALCON	489	3.16	1	40x1.10 water	142	512x512	510-540	80 MHz<br>500 photons
**Fig. 1C**	LysoTracker-Green	SP8-FALCON	489	3.16	1	40x1.10 water	142	512x512	510-540	80 MHz<br>500 photons
**Fig. 1C**	RhodB	SP8-FALCON	550	3.16	1	40x1.10 water	142	512x512	570-600	80 MHz<br>500 photons
**Fig. 1C**	BioTracker Orange Lysosome	SP8-FALCON	550	3.16	1	40x1.10 water	142	512x512	570-600	80 MHz<br>500 photons
**Fig. 1C**	SPY555-Actin	SP8-FALCON	550	3.16	1	40x1.10 water	142	512x512	570-600	80 MHz<br>500 photons
**Fig. 1C**	SPY555-DNA	SP8-FALCON	550	3.16	1	40x1.10 water	142	512x512	570-600	80 MHz<br>500 photons
**Fig. 1C**	SPY555-Tubulin	SP8-FALCON	550	3.16	1	40x1.10 water	142	512x512	570-600	80 MHz<br>500 photons
**Fig. 1C**	SPY620-Actin	SP8-FALCON	615	3.16	1	40x1.10 water	142	512x512	635-700	80 MHz<br>500 photons
**Fig. 1C**	SPY620-DNA	SP8-FALCON	615	3.16	1	40x1.10 water	142	512x512	635-700	80 MHz<br>500 photons
**Fig. 1C**	SiR700-Actin	SP8-FALCON	670	3.16	1	40x1.10 water	142	512x512	710-760	80 MHz<br>500 photons
**Fig. 1C**	SPY700-DNA	SP8-FALCON	670	3.16	1	40x1.10 water	142	512x512	710-760	80 MHz<br>500 photons
**Fig. 1C**	SiR700-Tubulin	SP8-FALCON	670	3.16	1	40x1.10 water	142	512x512	710-760	80 MHz<br>500 photons
**Fig2A**	MitoTracker-Green<br>LysoTracker-Green	SP8-FALCON	489	2.30	1	40x1.10 water	90	704x704	510-540	80 MHz<br>6 line accumu.
**Fig2B**	RhodB<br>SPY555-DNA	SP8-FALCON	550	2.70	1	40x1.10 water	102	600x600	570-600	80 MHz<br>10 line accumu.
**Fig2C**	SPY620-DNA	SP8-FALCON	615	4.06	1	40x1.10 water	112	400x400	635-700	80 MHz

	SPY620-Actin									10 line accumu.
**Fig2D**	SPY700-DNA<br>SiR700-Tubulin	SP8-FALCON	670	8.49	1	40x1.10 water	122	464x464	710-760	80 MHz<br>10 line accumu.
**Fig3B**	MitoTracker-Green<br>LysoTracker-Green	SP8-FALCON	489	2.54	1	40x1.10 water	114	840x840	510-540	80 MHz<br>10 line accumu.
**Fig3B**	Lyn11-SNAPf<br>MaP555-BG<br>SPY555-Actin	SP8-FALCON	550	2.54	1	40x1.10 water	114	840x840	570-600	80 MHz<br>10 line accumu.
**Fig3B**	CalR-HaloTag7-KDEL<br>β4Gal-T1-HaloTag11<br>MaP618-CA	SP8-FALCON	615	2.54	1	40x1.10 water	114	840x840	635-660	80 MHz<br>10 line accumu.
**Fig3B**	SPY700-DNA<br>SiR700-Tubulin	SP8-FALCON	670	2.54	1	40x1.10 water	114	840x840	710-760	80 MHz<br>10 line accumu.
**S1A**	MitoTracker-Green	SP8-FALCON	489	3.16	1	40x1.10 water	142	512x512	510-540	80 MHz<br>500 photons
**S1A**	LysoTracker-Green	SP8-FALCON	489	3.16	1	40x1.10 water	142	512x512	510-540	80 MHz<br>500 photons
**S1B**	MitoTracker-Red	SP8-FALCON	575	3.16	1	40x1.10 water	142	512x512	595-625	80 MHz<br>500 photons
**S1B**	LysoTracker-Red	SP8-FALCON	575	3.16	1	40x1.10 water	142	512x512	595-625	80 MHz<br>500 photons
**S1B**	SPY620-Actin	SP8-FALCON	615	3.16	1	40x1.10 water	142	512x512	635-700	80 MHz<br>500 photons
**S1B**	SPY620-DNA	SP8-FALCON	615	3.16	1	40x1.10 water	142	512x512	635-700	80 MHz<br>500 photons
**S1C**	RhodB	SP8-FALCON	550	3.16	1	40x1.10 water	142	512x512	570-600	80 MHz<br>500 photons
**S1C**	BioTracker Orange<br>Lysosome	SP8-FALCON	550	3.16	1	40x1.10 water	142	512x512	570-600	80 MHz<br>500 photons
**S1C**	SPY555-Actin	SP8-FALCON	550	3.16	1	40x1.10 water	142	512x512	570-600	80 MHz<br>500 photons
**S1C**	SPY555-DNA	SP8-FALCON	550	3.16	1	40x1.10 water	142	512x512	570-600	80 MHz<br>500 photons
**S1C**	SPY555-Tubulin	SP8-FALCON	550	3.16	1	40x1.10 water	142	512x512	570-600	80 MHz<br>500 photons
**S1C**	MitoTracker-Orange	SP8-FALCON	550	3.16	1	40x1.10 water	142	512x512	570-600	80 MHz<br>500 photons

**S1D**	SiR-DNA	SP8-FALCON	631	3.16	1	40x1.10 water	569	512x512	650-700	80 MHz<br>1,000<br>4 line accumu.
**S1D**	SiR-Actin	SP8-FALCON	631	3.16	1	40x1.10 water	569	512x512	650-700	80 MHz<br>1,000<br>4 line accumu.
**S1D**	SiR-Tubulin	SP8-FALCON	631	3.16	1	40x1.10 water	569	512x512	650-700	80 MHz<br>1,000<br>4 line accumu.
**S1E**	SiR700-Actin	SP8-FALCON	670	3.16	1	40x1.10 water	142	512x512	710-760	80 MHz<br>500 photons
**S1E**	SPY700-DNA	SP8-FALCON	670	3.16	1	40x1.10 water	142	512x512	710-760	80 MHz<br>500 photons
**S1E**	SiR700-Tubulin	SP8-FALCON	670	3.16	1	40x1.10 water	142	512x512	710-760	80 MHz<br>500 photons
**S2A**	MitoTracker-Green or LysoTracker-Green	SP8	*	3.16	1	40x1.10 water	142	512x512	*	Spectra
**S2B**	MaP618-CA or SPY620-DNA or SPY620-Actin	SP8	*	3.16	1	40x1.10 water	142	512x512	*	Spectra
**S2B**	LysoTracker-Red	SP8	*	3.16	1	40x1.10 water	142	512x512	*	Spectra
**S2C-D**	MaP555-CA or MaP555-BG or SPY555-DNA or SPY555-Actin or SPY555-Tubulin or Rhodamine B or Bio Tracker Orange Lysosome	SP8	*	3.16	1	40x1.10 water	142	512x512	*	Spectra
**S2E**	SPY700-DNA or SiR700-Actin or SiR700-Tubulin	SP8	*	3.16	1	40x1.10 water	142	512x512	*	Spectra
**S3**	SPY555-DNA	SP8-FALCON	550	3.16	1	40x1.10 water	142	512x512	570-600	80 MHz<br>500 photons
**S3**	SPY555-Tubulin	SP8-FALCON	550	3.16	1	40x1.10 water	142	512x512	570-600	80 MHz<br>500 photons
**S4A**	MitoTracker-Green	SP8-FALCON	489	2.30	1	40x1.10 water	90	704x704	510-540	80 MHz

	LysoTracker-Green									6 line accumu.
**S4B**	SPY620-DNA<br>SPY620-Actin	SP8-FALCON	615	4.06	1	40x1.10 water	112	400x400	635-700	80 MHz<br>10 line accumu.
**S4C**	SPY700-DNA<br>SiR700-Actin	SP8-FALCON	670	6.65	1	40x1.10 water	123	592x592	710-760	80 MHz<br>10 line accumu.
**S4D**	SPY700-DNA<br>SiR700-Tubulin	SP8-FALCON	670	8.49	1	40x1.10 water	122	464x464	710-760	80 MHz<br>10 line accumu.
**S5A**	Bio Tracker Orange Lysosome<br>SPY555-DNA	SP8-FALCON	550	2.85	1	40x1.10 water	101	568x568	570-600	80 MHz<br>10 line accumu.
**S5B**	SPY555-Actin<br>SPY555-DNA	SP8-FALCON	550	2.50	1	40x1.10 water	101	648x648	570-600	80 MHz<br>10 line accumu.
**S5C**	RhodB<br>SPY555-DNA	SP8-FALCON	550	2.70	1	40x1.10 water	102	600x600	570-600	80 MHz<br>10 line accumu.
**S5D**	SPY555-Tubulin<br>SPY555-DNA	SP8-FALCON	550	1.56	1	40x1.10 water	101	1040x1040	570-600	80 MHz<br>10 line accumu.
**S6A**	Bio Tracker Orange Lysosome<br>SPY555-DNA	SP8-FALCON	550	3.69	1	40x1.10 water	101	440x440	570-600	80 MHz<br>10 line accumu.
**S6B**	SPY555-Tubulin<br>SPY555-DNA	SP8-FALCON	550	9.85	1	40x1.10 water	100	400x400	570-600	80 MHz<br>10 line accumu.
**S6C**	Bio Tracker Orange Lysosome<br>SPY555-DNA	SP8-FALCON	550	4.06	1	40x1.10 water	101	400x400	570-600	80 MHz<br>10 line accumu.
**S6D**	SPY555-Tubulin<br>SPY555-DNA	SP8-FALCON	550	7.24	1	40x1.10 water	100	544x544	570-600	80 MHz<br>10 line accumu.
**S7A**	SPY555-DNA<br>CalR-Halo-KDEL<br>MaP555-CA	SP8-FALCON	550	2.25	1	40x1.10 water	101	728x728	570-600	80 MHz<br>10 line accumu.
**S7B**	SPY555-Actin<br>CalR-Halo-KDEL<br>MaP555-CA	SP8-FALCON	550	1.70	1	40x1.10 water	101	952x952	570-600	80 MHz<br>10 line accumu.
**S7C**	SPY555-DNA<br>CalR-SNAP-KDEL<br>MaP555-BG	SP8-FALCON	550	2.25	1	40x1.10 water	101	720x720	570-600	80 MHz<br>10 line accumu.
**S7D**	SPY555-Actin<br>CalR-SNAP-KDEL<br>MaP555-BG	SP8-FALCON	550	1.93	1	40x1.10 water	101	840x840	570-600	80 MHz<br>10 line accumu.

**S8**	SPY620-DNA<br>CalR-Halo-KDEL<br>MaP618-CA	SP8-FALCON	615	2.50	1	40x1.10 water	114	648x648	635-700	80 MHz<br>10 line accumu.
**S9A**	MitoTracker-Green<br>LysoTracker-Green	SP8-FALCON	489	1.96	1	40x1.10 water	90	824x824	510-540	80 MHz<br>10 line accumu.
**S9A**	SPY555-DNA<br>SPY555-Tubulin	SP8-FALCON	550	1.96		40x1.10 water	90	824x824	570-600	80 MHz<br>10 line accumu.
**S9B**	MitoTracker-Green<br>LysoTracker-Green	SP8-FALCON	489	1.83	1	40x1.10 water	90	888x888	510-540	80 MHz<br>10 line accumu.
**S9B**	SPY555-DNA<br>SPY555-Actin	SP8-FALCON	550	1.83	1	40x1.10 water	90	888x888	570-600	80 MHz<br>10 line accumu.
**S10**	MitoTracker-Green<br>LysoTracker-Green	SP8-FALCON	489	1.89	1	40x1.10 water	90	856x856	510-540	80 MHz<br>12 line accumu.
**S10**	SPY620-DNA<br>SPY620-Actin	SP8-FALCON	615	1.89	1	40x1.10 water	90	856x856	635-700	80 MHz<br>12 line accumu.
**S11A**	MitoTracker-Green<br>LysoTracker-Green	SP8-FALCON	489	2.01	1	40x1.10 water	90	776x776	510-540	80 MHz<br>10 line accumu.
**S11A**	CalR-Halo-KDEL<br>MaP555-CA<br>SPY555-Actin	SP8-FALCON	550	2.01	1	40x1.10 water	90	776x776	570-600	80 MHz<br>10 line accumu.
**S11A**	SPY700-DNA<br>SiR700-Tubulin	SP8-FALCON	670	2.01	1	40x1.10 water	90	776x776	710-760	80 MHz<br>10 line accumu.
**S11B**	MitoTracker-Green<br>LysoTracker-Green	SP8-FALCON	489	1.46	1	40x1.10 water	90	712x712	510-540	80 MHz<br>10 line accumu.
**S11B**	CalR-Halo-KDEL<br>MaP555-CA<br>SPY555-Actin	SP8-FALCON	550	1.46	1	40x1.10 water	90	712x712	570-600	80 MHz<br>10 line accumu.
**S11B**	SPY700-DNA<br>SiR700-Tubulin	SP8-FALCON	670	1.46	1	40x1.10 water	90	712x712	710-760	80 MHz<br>10 line accumu.
**S11C**	MitoTracker-Green<br>LysoTracker-Green	SP8-FALCON	489	2.00	1	40x1.10 water	90	1160x1160	510-540	80 MHz<br>10 line accumu.
**S11C**	CalR-Halo-KDEL<br>MaP555-CA<br>SPY555-Actin	SP8-FALCON	550	2.00	1	40x1.10 water	90	1160x1160	570-600	80 MHz<br>10 line accumu.
**S11C**	SPY700-DNA<br>SiR700-Tubulin	SP8-FALCON	670	2.00	1	40x1.10 water	90	1160x1160	710-760	80 MHz<br>10 line accumu.
**S12**	MitoTracker-Green<br>LysoTracker-Green	SP8-FALCON	489	3.14	1	40x1.10 water	122	680x680	510-540	80 MHz<br>10 Lin accumu

										every 2 min
**S12**	CalR-Halo-KDEL<br>MaP555-CA<br>SPY555-Actin	SP8-FALCON	550	3.14	1	40x1.10 water	122	680x680	570-600	80 MHz<br>10 Lin accumu<br>every 2 min
**S12**	SPY700-DNA<br>SiR700-Tubulin	SP8-FALCON	670	3.14	1	40x1.10 water	122	680x680	710-760	80 MHz<br>10 Lin accumu<br>every 2 min
**S13**	MitoTracker-Green<br>LysoTracker-Green	SP8-FALCON	489	2.54	1	40x1.10 water	114	840x840	510-540	80 MHz<br>10 line accumu.
**S13**	Lyn11-SNAPf<br>MaP555-BG<br>SPY555-Actin	SP8-FALCON	550	2.54	1	40x1.10 water	114	840x840	570-600	80 MHz<br>10 line accumu.
**S13**	CalR-HaloTag7-KDEL<br>β4Gal-T1-HaloTag11<br>MaP618-CA	SP8-FALCON	615	2.54	1	40x1.10 water	114	840x840	635-660	80 MHz<br>10 line accumu.
**S13**	SPY700-DNA<br>SiR700-Tubulin	SP8-FALCON	670	2.54	1	40x1.10 water	114	840x840	710-760	80 MHz<br>10 line accumu.
**Tab S2-3**	MitoTracker-Green	SP8-FALCON	489	3.16	1	40x1.10 water	142	512x512	510-540	80 MHz<br>500 photons
**Tab S2-3**	LysoTracker-Green	SP8-FALCON	489	3.16	1	40x1.10 water	142	512x512	510-540	80 MHz<br>500 photons
**Tab S2-3**	RhodB	SP8-FALCON	550	3.16	1	40x1.10 water	142	512x512	570-600	80 MHz<br>500 photons
**Tab S2-3**	BioTracker Orange<br>Lysosome	SP8-FALCON	550	3.16	1	40x1.10 water	142	512x512	570-600	80 MHz<br>500 photons
**Tab S2-3**	SPY555-Actin	SP8-FALCON	550	3.16	1	40x1.10 water	142	512x512	570-600	80 MHz<br>500 photons
**Tab S2-3**	SPY555-DNA	SP8-FALCON	550	3.16	1	40x1.10 water	142	512x512	570-600	80 MHz<br>500 photons
**Tab S2-3**	SPY555-Tubulin	SP8-FALCON	550	3.16	1	40x1.10 water	142	512x512	570-600	80 MHz<br>500 photons
**Tab S2**	LysoTracker-Red	SP8-FALCON	575	3.16	1	40x1.10 water	142	512x512	595-625	80 MHz<br>500 photons
**Tab S2-3**	SPY620-Actin	SP8-FALCON	615	3.16	1	40x1.10 water	142	512x512	635-700	80 MHz<br>500 photons
**Tab S2-3**	SPY620-DNA	SP8-FALCON	615	3.16	1	40x1.10 water	142	512x512	635-700	80 MHz<br>500 photons
**Tab S2-3**	SiR700-Actin	SP8-FALCON	670	3.16	1	40x1.10 water	142	512x512	710-760	80 MHz

										500 photons
**Tab S2-3**	SPY700-DNA	SP8-FALCON	670	3.16	1	40x1.10 water	142	512x512	710-760	80 MHz<br>500 photons
**Tab S2-3**	SiR700-Tubulin	SP8-FALCON	670	3.16	1	40x1.10 water	142	512x512	710-760	80 MHz<br>500 photons
**Tab S4**	H2B-SNAP<br>MaP555-BG	SP8-FALCON	550	3.16	1	40x1.10 water	142	512x512	570-600	80 MHz<br>500 photons
**Tab S4**	SNAP-LMNB1<br>MaP555-BG	SP8-FALCON	550	5.64	1	40x1.10 water	101	288x288	570-600	80 MHz<br>500 photons
**Tab S4**	SNAP<br>MaP555-BG	SP8-FALCON	550	3.16	1	40x1.10 water	142	512x512	570-600	80 MHz<br>500 photons
**Tab S4**	NES-SNAP<br>MaP555-BG	SP8-FALCON	550	3.16	1	40x1.10 water	142	512x512	570-600	80 MHz<br>500 photons
**Tab S4**	Tomm20-SNAP<br>MaP555-BG	SP8-FALCON	550	3.16	1	40x1.10 water	142	512x512	570-600	80 MHz<br>500 photons
**Tab S4**	COX8-SNAP<br>MaP555-BG	SP8-FALCON	550	3.16	1	40x1.10 water	142	512x512	570-600	80 MHz<br>500 photons
**Tab S4**	CalR-SNAP-KDEL<br>MaP555-BG	SP8-FALCON	550	3.16	1	40x1.10 water	142	512x512	570-600	80 MHz<br>500 photons
**Tab S4**	β4Gal-T1-SNAP<br>MaP555-BG	SP8-FALCON	550	3.16	1	40x1.10 water	142	512x512	570-600	80 MHz<br>500 photons
**Tab S4**	LAMP1-SNAP<br>MaP555-BG	SP8-FALCON	550	3.16	1	40x1.10 water	142	512x512	570-600	80 MHz<br>500 photons
**Tab S4**	SNAP-SKL<br>MaP555-BG	SP8-FALCON	550	7.69	1	40x1.10 water	142	512x512	570-600	80 MHz<br>500 photons
**Tab S4**	Lyn11-SNAP<br>MaP555-BG	SP8-FALCON	550	3.16	1	40x1.10 water	142	512x512	570-600	80 MHz<br>500 photons
**Tab S4**	Ig-κ-SNAP-PDGFR<br>MaP555-BG	SP8-FALCON	550	3.16	1	40x1.10 water	142	512x512	570-600	80 MHz<br>500 photons
**Tab S4**	CEP41-SNAP<br>MaP555-BG	SP8-FALCON	550	3.16	1	40x1.10 water	142	512x512	570-600	80 MHz<br>500 photons
**Tab S4**	Lifeact-SNAP<br>MaP555-BG	SP8-FALCON	550	3.16	1	40x1.10 water	142	512x512	570-600	80 MHz<br>500 photons

References

(1) Gibson, D. G.; Young, L.; Chuang, R. Y.; Venter, J. C.; Hutchison III, C. A.; Smith, H. O. Enzymatic Assembly of DNA Molecules up to Several Hundred Kilobases. *Nat. Methods* **2009**, *6* (5), 343–345.

(2) Frei, M. S.; Hoess, P.; Lampe, M.; Nijmeijer, B.; Kueblbeck, M.; Ellenberg, J.; Wadepohl, H.; Ries, J.; Pitsch, S.; Reymond, L.; Johnsson, K. Photoactivation of Silicon Rhodamines via a Light-Induced Protonation. *Nat. Commun.* **2019**, *10* (1), 4580.

(3) Sallin, O.; Reymond, L.; Gondrand, C.; Raith, F.; Koch, B.; Johnsson, K. Semisynthetic Biosensors for Mapping Cellular Concentrations of Nicotinamide Adenine Dinucleotides. *Elife* **2018**, *7*, e32638.

(4) Brun, M. A.; Tan, K. T.; Griss, R.; Kielkowska, A.; Reymond, L.; Johnsson, K. A Semisynthetic Fluorescent Sensor Protein for Glutamate. *J. Am. Chem. Soc.* **2012**, *134* (18), 7676–7678.

(5) Wilhelm, J.; Kühn, S.; Tarnawski, M.; Gotthard, G.; Tänzer, T.; Karpenko, J.; Mertes, N.; Xue, L.; Uhrig, U.; Halotag, T.; Wilhelm, J.; Ku, S.; Tarnawski, M.; Gotthard, G.; Tu, J.; Timo, T.; Karpenko, J.; Mertes, N.; Xue, L.; Uhrig, U.; Reinstein, J.; Hiblot, J.; Johnsson, K.; Kühn, S.; Tarnawski, M.; Gotthard, G.; Tünnermann, J.; Tänzer, T.; Karpenko, J.; Mertes, N.; Xue, L.; Uhrig, U.; Reinstein, J.; Hiblot, J.; Johnsson, K.; Kuehn, S.; Tarnawski, M.; Gotthard, G.; Tuennermann, J.; Taenzer, T.; Karpenko, J.; Mertes, N.; Xue, L.; Uhrig, U.; Reinstein, J.; Hiblot, J.; Johnsson, K.; Kühn, S.; Tarnawski, M.; Gotthard, G.; Tünnermann, J.; Tänzer, T.; Karpenko, J.; Mertes, N.; Xue, L.; Uhrig, U.; Reinstein, J.; Hiblot, J.; Johnsson, K. Kinetic and Structural Characterization of the Self-Labeling Protein Tags HaloTag7, SNAP-Tag, and CLIP-Tag. *Biochemistry* **2021**, *60* (33), 2560–2575.

(6) Moeyaert, B.; Holt, G.; Madangopal, R.; Perez-Alvarez, A.; Fearey, B. C.; Trojanowski, N. F.; Ledderose, J.; Zolnik, T. A.; Das, A.; Patel, D.; Brown, T. A.; Sachdev, R. N. S.; Eickholt, B. J.; Larkum, M. E.; Turrigiano, G. G.; Dana, H.; Gee, C. E.; Oertner, T. G.; Hope, B. T.; Schreiter, E. R. Improved Methods for Marking Active Neuron Populations. *Nat. Commun.* **2018**, *9* (1), 4440.

(7) Goedhart, J.; Von Stetten, D.; Noirclerc-Savoye, M.; Lelimousin, M.; Joosen, L.; Hink, M. A.; Van Weeren, L.; Gadella Jr, T. W. J.; Royant, A. Structure-Guided Evolution of Cyan Fluorescent Proteins towards a Quantum Yield of 93%. *Nat. Commun.* **2012**, *3*, 751.

(8) Falcón-Pérez, J. M.; Nazarian, R.; Sabatti, C.; Dell'Angelica, E. C. Distribution and Dynamics of Lamp1-Containing Endocytic Organelles in Fibroblasts Deficient in BLOC-3. *J. Cell Sci.* **2005**, *118* (22), 5243–5255.

(9) Malecki, M. J.; Sanchez-Irizarry, C.; Mitchell, J. L.; Histen, G.; Xu, M. L.; Aster, J. C.; Blacklow, S. C. Leukemia-Associated Mutations within the NOTCH1 Heterodimerization Domain Fall into at Least Two Distinct Mechanistic Classes. *Mol. Cell. Biol.* **2006**, *26* (12), 4642–4651.

(10) Rueden, C. T.; Schindelin, J.; Hiner, M. C.; DeZonia, B. E.; Walter, A. E.; Arena, E. T.; Eliceiri, K. W. ImageJ2: ImageJ for the next Generation of Scientific Image Data. *BMC Bioinformatics* **2017**, *18* (1), 529.

(11) Schindelin, J.; Arganda-Carreras, I.; Frise, E.; Kaynig, V.; Longair, M.; Pietzsch, T.; Preibisch, S.; Rueden, C.; Saalfeld, S.; Schmid, B.; Tinevez, J.-Y.; White, D. J.; Hartenstein, V.; Eliceiri, K.; Tomancak, P.; Cardona, A. Fiji: An Open-Source Platform for Biological-Image Analysis. *Nat. Methods* **2012**, *9* (7), 676–682.

(12) Origin(Pro), Version 2018b, OriginLab Corporation, Northampton, MA, USA.

(13) Wang, L.; Tran, M.; D'Este, E.; Roberti, J.; Koch, B.; Xue, L.; Johnsson, K. A General Strategy to Develop Cell Permeable and Fluorogenic Probes for Multicolour Nanoscopy. *Nat. Chem.* **2020**, *12*, 165–172.

(14) Lukinavičius, G.; Blaukopf, C.; Pershagen, E.; Schena, A.; Reymond, L.; Derivery, E.; Gonzalez-Gaitan, M.; D'Este, E.; Hell, S. W.; Gerlich, D. W.; Johnsson, K. SiR-Hoechst Is a Far-Red DNA Stain for Live-Cell Nanoscopy. *Nat. Commun.* **2015**, *6*, 8497.

(15) Lukinavičius, G.; Reymond, L.; D'Este, E.; Masharina, A.; Göttfert, F.; Ta, H.; Güther, A.; Fournier, M.; Rizzo, S.; Waldmann, H.; Blaukopf, C.; Sommer, C.; Gerlich, D. W.; Arndt, H.-D.; Hell, S. W.;

Johnsson, K. Fluorogenic Probes for Live-Cell Imaging of the Cytoskeleton. *Nat. Methods* **2014**, *11* (7), 731–733.

(16) Lukinavičius, G.; Reymond, L.; Umezawa, K.; Sallin, O.; D'Este, E.; Göttfert, F.; Ta, H.; Hell, S. W.; Urano, Y.; Johnsson, K. Fluorogenic Probes for Multicolor Imaging in Living Cells. *J. Am. Chem. Soc.* **2016**, *138*, 9365–9368.

(17) Frei, M. S.; Tarnawski, M.; Roberti, M. J.; Koch, B.; Hiblot, J.; Johnsson, K. Engineered HaloTag Variants for Fluorescence Lifetime Multiplexing. *Nat. Methods* **2022**, *19*, 65–70.
